# Anticancer and Antimicrobial Activities of New Cobalt and Zinc Complex-Derived Benzimidazole Containing Nitro or Methyl Groups

**DOI:** 10.1021/acsomega.5c08353

**Published:** 2026-02-09

**Authors:** Ozgur Yilmaz, Elif Apohan, Ozfer Yesilada, Ulkü Yılmaz, Hasan Küçükbay

**Affiliations:** † Department of Biology, 37520 Inonu University, Art and Science Faculty, Malatya 44280, TURKEY; ‡ Department of Engineering Basic Sciences, 531771 Malatya Turgut Özal University, Engineering and Natural Sciences Faculty, Malatya 44280, TURKEY; § Inonu University, Art and Science Faculty, Department of Chemistry, Malatya 44280, TURKEY

## Abstract

The cytotoxic effects of six newly synthesized 5(6)-methyl or 5(6)-nitrobenzimidazole-derived compounds that contain cobalt and zinc on nonsmall cell lung carcinoma (A549) and healthy lung epithelial (BEAS-2B) cell lines were evaluated by MTT, caspase-3, and Western Blotting methods. Cisplatin was also used as a reference compound. Compound **4** and compound **5** exhibited high cytotoxic activity against A549. While the IC_50_ value of cisplatin was 14.31 μM, IC_50_ values of compound **4** and compound **5** were 10.30 and 7.01 μM on A549 cells at 72 h, respectively. On the other hand, for BEAS-2B cells, the IC_50_ values of cisplatin, compound **4,** and compound **5** were 5.91, 90.13, and 51,68 μM, respectively. The results showed that although compounds **4** and **5** have high cytotoxicity on A549 cells, similar to cisplatin, they are less cytotoxic to BEAS-2B cells than cisplatin. The antibacterial and antifungal properties of the synthesized compounds were also investigated using minimum inhibitory concentration method. Their antimicrobial activities were tested against the Gram-negative bacteria *Escherichia coli* ATCC 25922 and *Pseudomonas aeruginosa* ATCC 27853, Gram-positive *Staphylococcus aureus* ATCC 29213, and yeast species *Candida albicans* ATCC 90028 and *Candida tropicalis*. The compounds showed notable antimicrobial activity against these pathogenic bacteria and yeasts.

## Graphical abstract

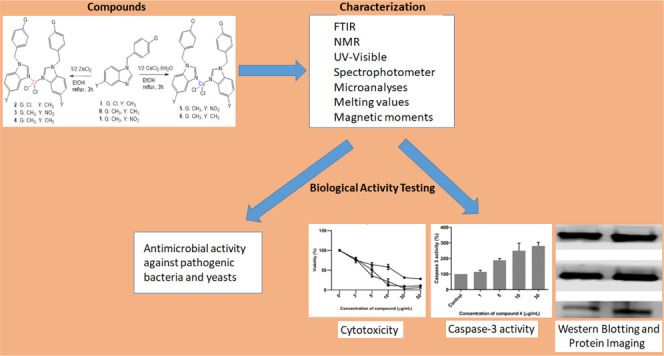

## 1 Introduction

It is estimated that approximately 9.7 million deaths were caused from cancer in 2022, with lung cancer being among the leading causes of cancer-related mortality.^1,2^ Cisplatin, a well-known metallodrug, is an effective chemotherapeutic agent widely used for cancer treatment, and many researchers continue to discover metal-containing compounds that could serve as alternative drugs.^3^ The development of more novel, effective, and less toxic drugs is crucial.^4^ Therefore, many scientists have focused on transition metals (particularly zinc and cobalt) for their potential in creating new metal-containing anticancer agents.^5,6^ These elements are essential for various enzymes.^7^ Zinc ions are important for cellular metabolism, growth, and development. They have positive effects on preneoplastic progression in rats.^8^ Moreover, zinc exhibits affinity for protein and DNA, but its binding affinity mostly depends on organic motifs.^9^ Metal compounds are very important for cancer treatment. Cobalt, a relatively nontoxic trace element, forms complexes that are promising agents for the treatment of cancer.^10,11^

Benzimidazole can be used an excellent scaffold for development of new drugs.^12^ Its electron-rich nitrogen heterocycles can accept or donate protons, facilitating weak interactions.^13^ Incorporating benzimidazole-derived moieties into the drug design may increase biological activities. Moreover, they are important pharmacophore in drug discovery with antitumor, antiulcer, antifungal, antibacterial, anthelmintic, anti-inflammatory, antitubercular, antihypertensive, and antiviral activities.^14–23^

Based on the results of the studies mentioned above, we examined the cytotoxic and antiapoptotic effects of newly synthesized benzimidazole compounds containing cobalt­(II) and zinc­(II) metal ions on the A549 cancer cell line and BEAS-2B healthy cell line. Additionally, antimicrobial effects of these benzimidazole compounds on Gram-positive (*Staphylococcus aureus*) and Gram-negative bacteria (*Escherichia coli* and *Pseudomonas aeruginosa*) and yeast strains *Candida albicans* and *Candida tropicalis* were investigated.

Although the main skeleton of these compounds is similar to that of the other benzimidazole complexes, they are novel, having different substituents in their benzimidazole ring systems. Biological activities of compounds that do not contain substituents on their homocyclic ring are widely documented in the literature.^23^ The present study aims to investigate the cytotoxic and antimicrobial properties of newly synthesized benzimidazole–metal complexes containing methyl and nitro substituents at the 5(6) position.

## 2 Materials and Methods

### 2.1 Chemicals and Equipment

The starting reagents used for the synthesis of new compounds were purchased from Aldrich or Merck.^1^HNMR (300 or 400 MHz) and ^13^CNMR (75 or 100 MHz) spectra were determined with a Bruker Avance FT NMR spectrometer using DMDO-*d*_6_ as a solvent. IR spectra were recorded with a PerkinElmer FTIR spectrophotometer. Microanalyses for the C, H, and N elements were performed with a LECO CHNS-932 analyzer. The electronic transition values of benzimidazole derivatives were measured with a UV–visible (PerkinElmer Lambda 35) spectrophotometer. Melting values of newly synthesized compounds (Figure 1) were assigned via an Electrothermal-9200 device. The magnetic moments of the paramagnetic compounds (**5** and **6**) were determined at RT using a Sherwood Scientific device.

**Figure 1:**
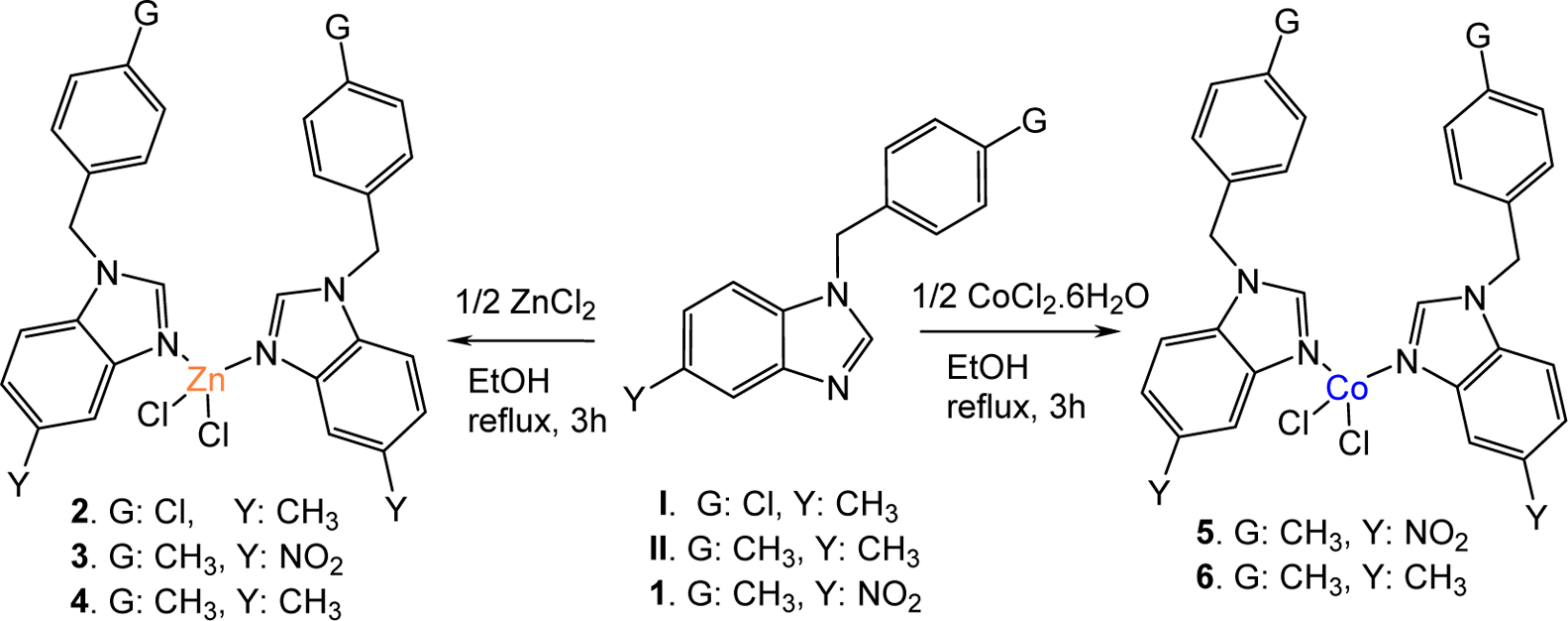
Synthesis of benzimidazole derivatives.

### 2.2 Synthesis

#### 2.2.1 Synthesis of 1-(4-Methylbenzyl)-5(6)-nitrobenzimidazole­(**1**)

A mixture of 5-nitrobenzimidazole (1.00 g, 6.14 mmol), *p*-methylbenzylbromide (1.14 g, 6.14 mmol), and KOH (0.34 g, 6.14 mmol) in 20 mL of ethanol was heated under reflux in a boiling flask for 5 h. After the completion of the reaction, the solvent was removed in vacuo. The resulting crude product was washed with water and then crystallized from ethanol/chloroform (1:1). Yield: 1.21 g, 74%, m.p.: 127–129 °C. Calcd for C_15_H_13_N_3_O_2_ (%): C, 67.40; H, 4.90; N, 15.72. Measured (%): C, 67.11; H, 4.82; N, 15.61. FTIR (cm^–1^): 2948 (w, C–H_aromatic_),1618 and 1591 (w, C–C_aromatic_), 1518 (s, NO_2_), 1469 (s, C–C_ring_), 1448 (s, C=N), 1393­(m), 1332­(s, NO_2_),1311 (s),1270 (w), 1236 (m), 1165 (m, C–H_benzene ring_), 1059 (m, C–H_in plane_), 1015 (m), 843 (m), 819 (s), 795 (s, C–H_out of plane benzene_), 754, 741, 733, (m, C–H_out of plane benzene_), 659 (m), imidazole ring), 472 (m), 422 (w). ^1^HNMR (400 MHz, δ): 8.78 and 8.73 (s, NC*H*N, 1H), 8.56–8.54 (m, Ar–*H*, 1H), 8.16–8.07 (m, Ar–*H*, 1H), 7.85 and 7.75 (2d, Ar–*H*, 1H, *J* = 9 Hz), 7.28–7.13 (m, Ar–*H*, 4H), 5.61 and 5.55 (s, benzylic–C*H*_2_, 2H), 2.24 (s, C*H*_3_, 3H) ppm.^13^CNMR (100 MHz, δ): 149.4 and 148.2 (N*C*HN), 147.9, 142.8, 137.9, 137.3, 133.1, 129.3, 127.5, 119.9, 118.0, 117.2, 115.7, 111.3, 107.8 (*C*_aromatic_), 47.8 (*C*H_2_), 20.6 (*C*H_3_) ppm. Compounds **I** and **II** were synthesized according to the literature using a similar method.^23–25^

#### 2.2.2 General Synthesis Procedure of Metal Complexes

The blend of ligand (compounds **I**, **II**, and **1**) (3.74 mmol), ZnCl_2_ or CoCl_2_·6H_2_O metal salt (1.87 mmol), and ethanol (20 mL) was reacted under reflux for 3 h. Then, the blend was cooled to RT and the impure product was filtered. After that, the product was purified by crystallization from ethanol/dimethylformamide (2:1).

##### 2.2.2.1 Synthesis of Dichlorobis­(1-(*p*-chlorobenzyl)-5­(6)-methyl-1H-benzimidazole-KN^3^)­zinc­(II) (**2**)

Yield: 1.04 g, 82%, mp 185–186 °C. Calcd for C_30_H_26_Cl_4_N_4_Zn (%): C, 55.46; H, 4.03; N, 8.62. Measured (%): C, 55.40; H, 4.10; N, 8.43. FTIR (cm^–1^): 3088 (w, C–H_aromatic_), 1671 (w, C–C_aromatic_), 1594­(w, C–C_aromatic_), 1515 (s, C=N), 1492 (m, ring C–C), 1383 (m), 1262 (m), 1194 (w, benzene ring C–H), 1071 (m, C–H_in plane_), 1013 (m), 892 (w, C–H_out of plane imidazole_), 841 (s), 803 (s), 792 (s), 773 (s, C–H_out of plane benzene_), 732 (s, C–H_out of plane benzene_), 676 (w, imidazole ring), 424 (s, Zn–N). ^1^HNMR (400 MHz, δ): 8.62 (s, NC*H*N, 2H), 7.58–7.11 (m, Ar–*H*, 14H), 5.56 (s, benzylic–C*H*_2_, 4H), 2.39 (s, C*H*_3_, 6H) ppm. ^13^C NMR (100 MHz, δ): 144.9 (N*C*HN), 135.9, 133.0, 129.9, 132.0, 131.0, 129.2, 125.3, 118.9, 111.4 (*C*_aromatic_), 47.7 (*C*H_2_), 21.6 (*C*H_3_) ppm.

##### 2.2.2.2 Synthesis of Dichlorobis­(1-(*p*-methylbenzyl)-5­(6)-nitro-*1H*-benzimidazole-KN^3^)­zinc­(II) (**3**)

Yield: 1.00 g, 78%, mp 198–200 °C. Calcd for C_30_H_26_Cl_2_N_6_O_4_Zn (%): C, 53.71; H, 3.91; N 12.53. Measured (%): C, 53.95; H, 3.77; N, 12.74. FTIR (cm^–1^): 3089 (w, C–H_aromatic_), 2974 (w, C–H_aliphatic_), 1622 (w, C–C_aromatic_), 1602 (w, C–C_aromatic_), 1529 (s, NO_2_), 1506 (m, C=N), 1494 (w, C–C_ring_), 1478 (w), 1392 (w), 1379 (w), 1345 (s, NO_2_), 1294 (w), 1188 (w, benzene ring C–H), 1087 (w, C–H_in plane_), 1067 (w), 881 (w, C–H_out of plane imidazole_), 826 (m), 794 (s, C–H_out of plane benzene_), 756 (m, C–H_out of plane benzene_), 733 (s, C–H)_out of plane benzene_, 662 (w, imidazole ring), 470 (m), 426 (s, Zn–N). ^1^HNMR (300 MHz, δ): 8.91 and 8.87 (s, NC*H*N, 2H), 8.66–8.60 (m, Ar–*H*, 2H), 8.19 and 7.79 (2d, Ar–*H*, 4H, *J* = 9 Hz), 7.30–7.13 (m, Ar–*H*, 8H), 5.66 and 5.59 (s, benzylic–C*H*_2_, 4H), 2.23 (s, CH_3_, 6H) ppm. ^13^CNMR (75 MHz, δ): 149.4, 148.5 (N*C*HN), 146.6, 143.1, 141.5, 137.6, 137.4, 132.8, 129.4, 127.5, 119.6, 118.5, 117.8, 115.4, 111.8, 108.2 (*C*_aromatic_), 48.0 and 47.9 (*C*H_2_), 20.6 (*C*H_3_) ppm.

##### 2.2.2.3 Synthesis of Dichlorobis­(1-(*p*-methylbenzyl)-5­(6)-methyl-*1H*-benzimidazole-KN^3^)­zinc­(II) (**4**)

Yield: 1.06 g, 82%, mp 217–218 °C. Calcd for C_32_H_32_Cl_2_N_4_Zn (%): C, 63.12; H, 5.30; N 10.74. Measured (%): C, 62.92; H, 5.18; N, 10.55. FTIR (cm^–1^): 3099 (CH_aromatic_), 2915 (CH_aliphatic_), 1672 (w), 1623 (w), 1591 (w), 1557 (m), 1508 (s, C=N), 1446 (w), 1383 (w),1263 (s), 1184 (m, C–H_benzene ring_), 1115 (w), 1022 (w, C–H_in plane_), 818, 798 (s, C–H_out of plane benzene_), 775 (s, C–H_out of plane benzene_), 720 (s, C–H_out of plane benzene_), 636 (m, imidazole ring), 470 (s), 426 (s, Zn–N). ^1^HNMR (400 MHz, δ): 8.85 and 8.81 (s, NC*H*N, 2H), 8.62–7.79 (m, Ar–*H* benzimidazole, 6H), 7.29–7.16 (m, Ar–*H* benzyl, 8H), 5.65 and 5.58 (s, benzylic–C*H*_2_, 4H), 2.26 (s, C*H*_3benzimidazole_, 6H) 2.09 (s, C*H*_3_ benzyl, 6H) ppm. ^13^CNMR (100 MHz, δ): 149.9 and 148.9 (N*C*HN), 143.5, 138.3, 137.9, 133.5, 129.8, 128.0, 120.2, 118.8, 118.1, 116.1, 112.2, 108.5 (*C*_aromatic_), 48.5 and 48.3 (*C*H_2_), 31.2 (*C*H_3benzimidazole_), 21.1 (*C*H_3benzyl_) ppm.

##### 2.2.2.4 Synthesis of Dichlorobis­(1-(*p*-methylbenzyl)-5­(6)-nitro-*1H*-benzimidazole-KN^3^)­cobalt­(II) (**5**)

Yield: 0.99 g, 80%, mp 183–185 °C. Calcd for C_30_H_26_Cl_2_CoN_6_O_4_ (%): C, 54.23; H, 3.94; N, 12.65. Measured (%): C, 54.15; H, 3.78; N, 12.90. FTIR (cm^–1^): 3088 (w, C–H_aromatic_), 1708 (w), 1622 (w, C–C_aromatic_), 1598 (w, C–C_aromatic_), 1528 (s, NO_2_), 1505 (s, C=N), 1447 (w), 1387 (w), 1344 (s, NO_2_), 1285 (m), 1186 (m, C–H_benzene ring_), 1120 (w), 1069 (w, C–H_in plane_), 1055 (w), 954 (w), 907 (w, C–H_out of plane imidazole_), 881 (w), 793 (s, C–H_out of plane benzene_), 756 (s, C–H_out of plane benzene_), 733 (s, C–H_out of plane benzene_), 658 (m, imidazole ring), 425 (Co–N), μeff: 5.19 (B.M.), λ_max_(nm)_d–d_: 688.

##### 2.2.2.5 Synthesis of Dichlorobis­(1-(*p*-methylbenzyl)-5­(6)-methyl-*1H*-benzimidazole-KN^3^)­cobalt­(II) (**6**)

Yield: 1.09 g, 85%, mp 223–224 °C. Calcd for C_32_H_32_Cl_2_CoN_4_ (%): C, 63.80; H, 5.35; N, 9.30. Measured (%): C, 63.50; H, 5.22; N, 9.18. FTIR (cm^–1^): 2922 (w, C–H_aromatic_), 1671 (w), 1512 (s, C=N), 1443 (m, C–C_ring_), 1330 (m), 1264 (w), 1187 (s, C–H_benzene ring_), 1020 (w), 812(s), 797 (s, C–H_out of plane benzene_), 720 (m, C–H_out of plane benzene_), 623 (m, imidazole ring), 432 (m), 422 (s, Co–N), μeff: 5.31­(B.M.), λ_max_(nm)_d–d_: 677.

### 2.3 Cell Lines and Culture Conditions

Human nonsmall cell lung carcinoma (A549) and healthy human bronchial epithelial (BEAS-2B) cell lines were used in this study. Throughout the study, the cells were kept alive by incubating them at 37 °C in 75 cm^2^ culture flasks containing Dulbecco’s modified Eagle’s medium with 10% fetal bovine serum and 1% penicillin/streptomycin.^26,27^

### 2.4 MTT Assay for Evaluation of the Cytotoxicity of the Compounds and Cisplatin

Dimethyl sulfoxide (DMSO) was used to prepare the stock solutions of the benzimidazole metal complexes, and further dilutions were prepared using fresh culture medium (DMSO concentration in the final culture medium was <0.1%).^25^ Cytotoxicity of the compounds was investigated by using the MTT cell proliferation assay. Five different concentrations of each compound were tested to determine the IC_50_ values. First, 5 × 10^3^ cells were cultivated in 96-well plates containing 100 μL of medium for 24 h, and then five different concentrations of the compounds were added. These were incubated at 37 °C in a 5% CO_2_ incubator for 24, 48, and 72 h. After that, 20 μL of MTT prepared in DPBS was added into each well and the plates were further incubated for 2 h. After the supernatant with thiazole was removed from the plates, 100 μL of DMSO was added into each well, and they were shaken at 150 rpm for 5 min in the dark at room temperature. Then, the formazan crystals were homogenized, and the plates were read at 570 nm. IC_50_ values of the compounds and cisplatin at 24, 48, and 72 h were calculated using the Microsoft Office Excel program. Standard deviation logarithmic slope graphs of the compounds were created by using the GraphPad program. Experiments were carried out in 12 replicates, and the results were expressed as the mean ± SD.

### 2.5 Apoptotic Activity Studies

Apoptotic activities of compounds and cisplatin were investigated by monitoring the caspase-3 activation and determining the protein expression by the Western Blot technique.

#### 2.5.1 Caspase-3 Activity Studies

BioAssay Systems Caspase-3 kit was used in this study. In this test, a specific substrate, N-Ac-DEVD-AFC, forms a highly fluorescent product through the activation of caspase-3. This fluorescence intensity is proportional to the caspase-3 activity.

A549 and BEAS-2B cell lines were cultivated in 6-well plates with 2.5 mL of medium per well. These cell lines were treated with four different concentrations (3.32, 16.61, 33.21, and 99.63 μM) of cisplatin and compounds. For caspase-3 activity analysis, 50 μL of cell lysate was added to the wells of the 96-well plates, and then 100 μL of reagent was added to each assay well. These plates were incubated at 37 °C for 60 min in the dark. Then, caspase-3 enzyme activity of the control group, compounds, and cisplatin-treated cells at 72 h was determined by measuring the absorbance at 405 nm using an ELISA reader. Total protein amount was also calculated by the Bradford method. The caspase-3 activities were calculated as percentages based on the effect of the compounds, and the graphs were performed.^28,29^ Experiments were carried out in three replicates, and the results were expressed as mean ± SD.

#### 2.5.2 Western Blotting and Protein Imaging

Protein imaging was performed using semidry blotting technique. The expression levels of β-actin, caspase-3, and p53 proteins were examined. Compounds and cisplatin were administered in a single dose to each cell line. The applied concentrations were determined based on the 72 h IC_50_ values obtained from the MTT cytotoxic activity assay results. Compounds **4**, **5,** and cisplatin were applied at a concentration of 16.61 μM to the A549 and BEAS-2B cell lines.

For Western Blotting, cells were seeded into 6-well plates as 1 × 10^5^ cells per well using three wells for each studied sample. Then, the compounds were added to these wells containing cells, and they were incubated in an incubator for 72 h. After that, cell pellets were obtained by discarding the supernatant after centrifugation. After cell lysis, the total protein concentrations were measured using the Bradford method. The samples adjusted to equal protein concentrations were loaded into the gel wells and electrophoresed. After running the proteins, the gels were carefully placed on the blotting membranes (PVDF membrane), and the proteins were transferred onto membranes. Then, these membranes were incubated with primary antibodies. Primary antibody application was carried out at +4 °C for 24 h at 70–80 rpm, and after that, the secondary antibodies were applied. ECL was added to the washed membranes and left for about 5 min. The proteins were visualized by imaging device (Licor Image Studio Digits).^30^

### 2.6 Microorganisms Used

Gram-negative bacteria *E. coli* ATCC 25922 and *P. aeruginosa* ATCC 27853, Gram-positive bacterium *S. aureus* ATCC 29213, and yeast species *C. albicans* ATCC 90028 and *C. tropicalis* were used to determine the antimicrobial activity of the compounds. While bacteria were incubated in a nutrient agar medium at 37 °C for 24 h, yeasts were incubated in a Sabouraud Dextrose agar medium for 48 h. The antimicrobial effects of the compounds were determined by their minimum inhibitory concentration (MIC) values.

### 2.7 Antimicrobial Activity Testing

Mueller–Hinton broth and RPMI-MOPS broth media were used as growth media for bacteria and yeasts, respectively. Appropriate amounts of these broth media were added to the wells of 96-well plates. The compounds were dissolved in DMSO and then distributed into these wells in decreasing concentration by serial dilution method. Microorganisms prepared based on the McFarland standard were reconstituted under sterile conditions and transferred into each well in an appropriate amount. Following incubation for 24 h for bacteria and 48 h for yeasts, MIC values were determined. Gentamicin and fluconazole antibiotics were used as positive controls. The antimicrobial activity of the compounds on bacteria and yeasts was determined based on their MIC values.^31,32^ The study was conducted in three replicates.

## 3 Results and Discussion

### 3.1 Synthesis and Characterization of the Benzimidazole Compounds

The 1-substituted benzimidazole compounds were synthesized according to the literature data (I and II). Compound **1** was synthesized in this study as a novel compound. Five new zinc­(II)-benzimidazole and cobalt­(II)-benzimidazole complexes were synthesized (Figure 1) and their structural characterizations were performed using these derivatives (compounds **2**–**6**).

In our previous studies on benzimidazole-metal complexes, X-ray structural analyses confirmed that the 1-substituted benzimidazole ligand coordinates to the metal via the nitrogen atom at position 3. Therefore, it is anticipated that, in the structures presented in Figure 1, the 1-substituted benzimidazole ligands coordinate to the metal through the nitrogen atom at position 3.^33–35^ The other spectral data are also consistent with those of previous similar studies. The percent yields of the synthesized compounds ranged from 74 to 85%. The structures of the compounds were elucidated using various analytical techniques, and the obtained spectral data were consistent with the previously reported literature data.^26^ The chemical shift of the proton at the second position of the benzimidazole ring (NC*H*N) in ligand **1** was shifted from 8.78 and 8.73 to 8.91 and 8.87 ppm in zinc­(II) complex (3). Similarly, the chemical shifts of the CH_2_ group were observed from 5.61 and 5.55 to 5.66 and 5.59 ppm in ^1^H NMR spectra. As expected, these values shifted to low area after bonding to metal. Because of the 5(6) tautomerization of the benzimidazole ligand, we observed more peaks than expected as indicated in the literature.^36^ In ^13^C NMR spectra of compounds **1** and **3**, specific chemical shift values of NC*H*N and CH_2_ groups were observed at 149.4 and 148.2, 149.4 and 148.5, and 47.8 and 48.0 ppm. The ν­(C=N) stretching frequencies of imino groups in the benzimidazole rings appeared at 1489, 1515, 1506, 1508, 1505, and 1512 cm^–1^ for compounds **1**-**6,** respectively. Aromatic C–H stretches were observed among the 3089–3043 cm^–1^. Additionally, N–O stretches belonging to the NO_2_ groups of compounds **1**, **3**, and **5** were detected at 1518 and 1332, 1530 and 1345, and 1528 and 1344 cm^–1^, respectively. In addition, the metal nitrogen bond peaks belonging to the complexes were observed in the range of 422–426 cm^–1^. Due to high spin electron configuration of cobalt, the magnetic moment values for cobalt complexes (**5** and **6**) were measured as 5.19 and 5.31 μ_B_ at RT. These spectral data are consistent with the literature reports as the compounds possess similar functional groups.^26,36,37^ The UV–visible spectral data are presented in Table 1. The lower electronic absorption bands (266–373 nm) detected for compounds **1**–**6** correspond to π → π* and *n* → π* transitions. The cobalt­(II) complexes (**5** and 6) displayed absorption bands in the visible region at 688 and 677 nm, respectively, which are attributed to d–d transitions. In our previous studies, it was shown that benzimidazole complexes synthesized using CoCl_2_ and ZnCl_2_ metal salts prefer the tetrahedral structure, in which 2 mol of benzimidazole and 2 mol of chlorine are bonded to the metal.^26,37^

**Table 1:** UV–Visible Spectral Bands of the Compounds and Magnetic Moment Values of the Cobalt Complexes

compound	electronic absorption bands λ_max_ (nm)		magnetic moment μ_eff (B.M.)_
	intraligand and charge transfer bands	d–dbands	
**1**	294, 277		
**2**	298, 277, 373		
**3**	293, 282, 279		
**4**	363, 310, 266		
**5**	294, 283, 279	688	5.19
**6**	364, 300, 293	677	5.31

These compounds are newly synthesized compounds. They have differences from the other benzimidazole compounds. For example, compared with the study reported by Apohan et al. in 2017, the most significant difference of the present work lies in the incorporation of electron-donating methyl and electron-withdrawing nitro substituents at the 5-position of benzimidazole in the newly synthesized compounds.^23^ In comparison with a similar skeletal framework article published by Yılmaz and Kucukbay in 2022, the substituents at the 5-position were retained, whereas the para-substitution at the 1-position of benzimidazole was modified by incorporating an electron-donating methyl group.^25^ Although there is a general resemblance in the skeletal structure, the diversity of substituents has been increased with the aim of contributing to filling the existing gap in the literature through the synthesis of new compounds and the exploration of their biological properties.

### 3.2 Biological Activity

#### 3.2.1 Cytotoxicity Studies

MTT studies demonstrated that two of the six compounds had pronounced cytotoxic activities. While compounds **4** and **5** exhibited high cytotoxic activity on the A549 cell line, they showed low cytotoxic activity on the BEAS-2B cell line, compared to cisplatin (Table 2). According to the 72 h IC_50_ values, compound **4** exhibited IC_50_ of 10.30 μM and 90.13 μM on A549 and BEAS-2B cells. Similarly, the IC_50_ values of compound **5** on A549 and BEAS-2B cells were 7.01 and 51.68 μM, respectively. Considering that cisplatin displayed a high IC_50_ value of 14.31 μM on A549 cells and a lower IC_50_ value of 5.91 μM on BEAS-2B healthy cells, these compounds seem to be compatible with the goals of anticancer drug development. The selective cytotoxicity of compounds **4** and **5** on cancer cells relative to healthy cells makes them candidate compounds for further applications.

**Table 2:** IC_50_ (μM) Values (Mean ± SD) of Six Benzimidazole-Co­(II) and -Zn­(II) Complexes on A549 and BEAS-2B Cells

	A549			BEAS-2B		
	24. hour	48. hour	72. hour	24. hour	48. hour	72. hour
**Compound 1**	>374,12 ± 0.961	255,26 ± 0.295	193,27 ± 0.665	229,00 ± 0.231	204,24 ± 0.183	167,76 ± 0.954
**Compound 2**	67,12 ± 0.001	38,43 ± 0.021	23,27 ± 0.049	60,34 ± 0.190	30,38 ± 0.150	39,08 ± 0.127
**Compound 3**	100,94 ± 0.113	35,83 ± 0.125	29,02 ± 0.031	45,20 ± 0.055	33,43 ± 0.055	34,10 ± 0.095
**Compound 4**	152,73 ± 0.100	74,64 ± 0.160	10,30 ± 0.634	101,64 ± 0.174	90,18 ± 0.293	90,13 ± 0.446
**Compound 5**	60,62 ± 0.269	61,50 ± 0.001	7,01 ± 4.254	90,50 ± 0.076	70,05 ± 0.198	51,68 ± 0.078
**Compound 6**	12,70 ± 0,03	6,77 ± 0.131	4,99 ± 0.487	18,31 ± 0.156	7,88 ± 0.197	5,01 ± 1.147
Cisplatin	53,13 ± 0.136	16,94 ± 0.132	14,31 ± 0.152	14,61 ± 0.202	7,37 ± 0.096	5,91 ± 0.222

For several decades, research groups have focused on the synthesis and application of new metal-based compounds with cytotoxic activity instead of the platinum-based compounds having side effects. In this context, various transition metal complexes such as those incorporating Fe, Co, Zn, Cu, Ti, Zr, Ru, Sn, Rh, Pd, Ag, and Au have been prepared and their activities have been determined. Metal-based complexes are potential anticancer agents due to their excellent selectivity. Research continues worldwide to develop effective anticancer agents.^38,39^ Suliman et al. (2023) reported that metal-based nanoparticles could be used to enhance the efficacy of metal ion-containing anticancer agents.^40^ Gai et al. (2023) stated that Cu­(II), Zn­(II), and Mn­(II) metal complexes with 5-chloro-2-N-(2-quinolylmethylene) aminophenol ligand have greater cytotoxic activity against A549 cancer cells than cisplatin, with copper and zinc complexes showing higher activity compared to the manganese complex.^41^ Teran et al. (2023) demonstrated the cytotoxic activity of sterically hindered dirutenium complexes.^42^ Similarly, Zhang et al. (2022) studied the ruthenium complexes and reported that although ligands enhance cytotoxic activity, the underlying cause of the activity is the metal atom.^43^ In other studies, conducted on this topic, Jiang et al. (2024) reported that Au-, Zn-, Pt-, Ru-, Cu-, Ni-, Co-, and Fe-centered complexes with Schiff-base ligands have high cytotoxic activity against lung cancer, with copper complexes showing the best cytotoxic activity.^44^ Moghadam et al. (2024) emphasized that the zinc complexes are much more noteworthy compounds in the development of anticancer agents due to their low toxicity compared to some metal-centered complexes.^45^ Additionally, it was reported that metal complexes containing selenium show increased anticancer efficacy.^46^ Suárez-Moreno et al. (2022) demonstrated that square planar metal complexes containing benzimidazole ligands had higher anticancer activity compared to tetrahedral complexes, and that this activity was particularly enhanced when the metal center was a second- or third-row element.^47^

Inhibition of the proliferation of cancer cells by benzimidazole derivative compounds containing Cu­(II), Zn­(II), Ni­(II), and Ag­(I) was previously reported.^48^ In our study, compound **6** also displayed high cytotoxicity in A549 cells. However, it also showed high cytotoxic activity on healthy BEAS-2B cells. The other three compounds (compounds **1**, **2,** and **3)** had a limited cytotoxic effect on A549 cells with IC_50_ values in the range of 23.27–193.27 μM (Table 2).

MTT studies showed that compounds **4** and **5** have a higher cytotoxic activity on A549 cells compared with the other four compounds. On the other hand, compound 6 had also high cytotoxicity on A549 and, however, it showed cytotoxic activity on BEAS-2B cells. Therefore, caspase-3 activity studies were conducted only with these two compounds. For comparison and as a control, the effect of cisplatin on caspase-3 activity was also investigated. Based on the 72 h IC_50_ values, four different concentrations (3.32, 16.61, 33.21, and 99.63 μM) were used for caspase-3 activity determination. As shown in Figure 2, the cisplatin treatment significantly increased the caspase-3 activity of A549 and BEAS-2B cells. More importantly, cisplatin caused a greater increase in the caspase-3 activity of BEAS-2B cells than A549 cells, especially at low concentrations. This is important for evaluating the effects of benzimidazole compounds studied. On the other hand, compound **4** showed a lower effect on activation of caspase-3 in BEAS-2B cells than in A549 cells, especially at 16.61–99.63 μM. Compounds **4** and **5** minimally induced the caspase-3 activity of BEAS-2B cells. Especially, the difference detected when 33.21 μM concentration used appears to be more prominent. While cisplatin induced the caspase-3 activity of A549 cells at a 16.61 μM concentration, it highly induced the caspase-3 activity of BEAS-2B cells at the same concentration. However, the caspase-3 activities detected in the BEAS-2B cells are lower than cisplatin when compounds **4** and **5** are used at a 16.61 μM concentration. Various studies showed that benzimidazole-based compounds initiate the apoptotic pathway by promoting the activation of caspase-3 in cells. It has been reported that these compounds trigger caspase-3 activity through several ways, such as altering mitochondrial membrane permeability, destabilizing DNA, activating the JNG signaling pathway, and inactivating topoisomerase I.^49–51^ Caspase-3 is an important enzyme that is activated during apoptosis. Therefore, the increased enzymatic activity and protein expression of caspase 3 demonstrate its important role in apoptotic cell death. Moreover, p53 is also an important regulator of the apoptotic pathway. Therefore, increases in caspase 3 activity and caspase 3 and p53 expression show the apoptotic mechanism in cell death.

**Figure 2:**
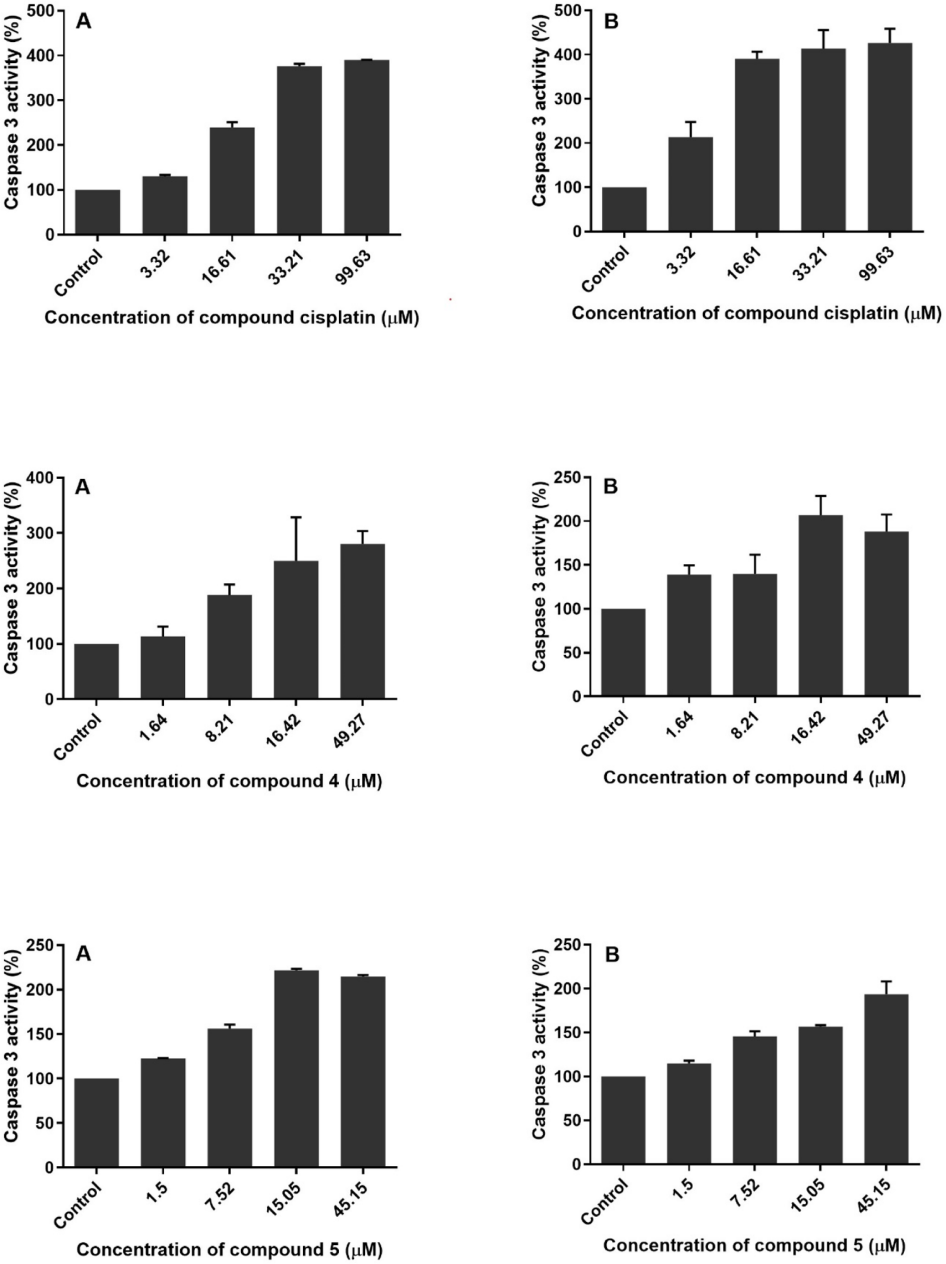
Caspase 3 activities of A549 (A) and BEAS-2B (B) cells treated with cisplatin, compound **4,** and compound **5** (exposure time: 72 h; concentrations: 0–99.63 μM). Data are presented as mean ± SD.

Generally, the biological activities of compounds without substituents on a homocyclic ring have been well documented in the literature.^23^ The aim of this study is to investigate the cytotoxic properties of new derivatives of benzimidazole-metal complexes containing methyl and nitro substituents at the 5(6) position. After examining the cytotoxic properties of derivatives with a 5(6)-nitro or methyl substituent and an electron-withdrawing halogen (Cl, Br) at the para position of the benzyl group at position 1,^26^ this study was conducted to investigate whether there would be any change in the cytotoxic properties of derivatives that possess a 5(6)-nitro or methyl substituent but contain an electron-donating methyl group at the para position of the benzyl moiety. Structure–activity relationships showed that the cobalt-containing paramagnetic benzimidazole complex (Y= NO_2_, G = Me) displays a lower cytotoxicity against healthy cells compared with cisplatin. The diamagnetic benzimidazole-metal (Zn) complex also showed high cytotoxic activity on A549 cells for derivatives with an electron-donating methyl substituent at the 5(6) position (compound **4**), while its cytotoxicity against healthy cells is found to be lower.

#### 3.2.2 Western Blotting Studies

Western blotting studies were performed to determine how compounds **4** and **5** affect the expression of some proteins involved in the apoptosis signaling pathway. Their effects on the expression levels of caspase-3 and p53 proteins, important proteins in the apoptosis pathway, were investigated in the A549 and BEAS-2B cell lines. The expression of β-actin, a reference protein, was also monitored. Cisplatin was also studied as a reference compound for comparison.

Figure 3 shows β-actin, caspase-3, and p53 protein expression levels detected in A549 and BEAS-2B cells treated with compounds **4** and **5**. Consistent with the results of the MTT and caspase-3 activity assay, compounds **4** and **5** had limited effect on BEAS-2B healthy cells compared with cisplatin. The caspase-3 and p53 protein expression levels showed that these compounds are more effective on the A549 cells.

**Figure 3:**
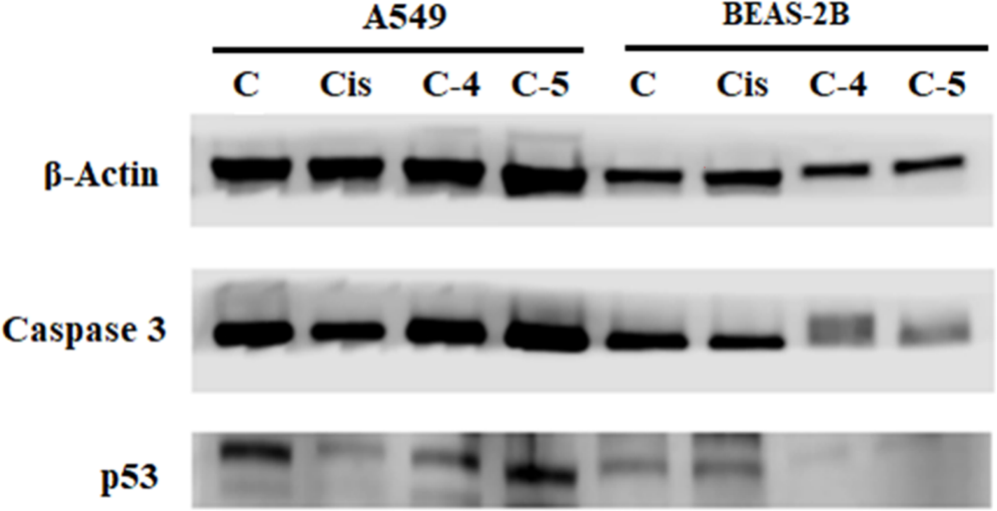
Western blot analysis of β-actin, caspase-3, and p53 protein expression levels in A549 and BEAS-2B cells treated with cisplatin, compound **4,** and compound **5** (C: control, Cis: cisplatin, C-4: compound **4**, C-5: compound **5**) (exposure time: 72 h; concentrations: 16.61 μM).

Several benzimidazole derivative compounds have been reported to exert cytotoxic effects on cancer cells by triggering an apoptotic pathway. In these studies, ATP assays, flow cytometry, Western blotting, and caspase-3/7 analyses were performed to evaluate the effects of the benzimidazole derivatives on cell proliferation, cell cycle progression, and apoptosis. Their results demonstrated that these compounds inhibited the growth of cancer cells, including HepG2 (hepatocellular carcinoma cell line) and cervical cancer HEp-2 (HeLa derivative) cell lines. Flow cytometry, Western blot, and caspase-3/7 activity assays indicated that benzimidazole derivatives induce the G1 phase cell cycle arrest, accompanied by increased expression of p53 (Ser15) and p21.^52^

#### 3.2.3 Antimicrobial Studies

The antibacterial and antifungal activities of these newly synthesized six compounds were tested by the MIC method.^53^ The antimicrobial activity results of the compounds are presented in Table 3, with the standard reference compounds fluconazole and gentamicin. While some compounds showed limited antimicrobial activity, others showed high antimicrobial activity. Compounds **1** and **3** displayed low antimicrobial activity on microorganisms. Among these compounds, compounds **2**, **4**, **5**, and **6** showed high antifungal activity against yeasts, with MIC values between 12.5 and 25 μg/mL for *C. albicans* and *C. tropicalis*. On the other hand, all compounds showed weak antimicrobial activity against *E. coli*. Compound **2** showed the highest antibacterial activity on *P. aeruginosa* and *S. aureus* with MIC values of 12.5 and 50 μg/mL, respectively. The lowest antimicrobial activity was determined against Gram-negative bacterium *E. coli* that has a two-layered cell wall with a thin peptidoglycan layer and the outer membrane. The outer membrane may reduce the antimicrobial effect of the compounds on *E. coli* by preventing their passage of them. They showed the highest antimicrobial activity on yeasts. According to the MIC test results, the 5(6)-methylbenzimidazole ligand in the zinc complex bearing a *p*-chlorobenzyl group was determined as the most effective antibacterial compound. In addition, cobalt complexes with *p*-methylbenzyl-substituted 5­(6)-methyl and 5(6)-nitrobenzimidazole ligands showed higher antimicrobial activity against *C. albicans* and *C. tropicalis*. It was reported that benzyl-substituted benzimidazole zinc complexes containing chlorine and methyl groups, especially in the para position, showed remarkable inhibitory activities against bacterial and fungal species.^23^ Additionally, it was also stated that 5(6)-methylbenzimidazole ligand and their zinc and cobalt complexes, especially those containing a chlorine group in the para position, possess high antimicrobial activity.^26^ Previous studies on antimicrobial activities of cobalt-, zinc-, and nickel-based benzimidazole compounds against *Escherichia faecalis* ATCC 29212, *S. aureus* ATCC 29213, *E. coli* ATCC 25922, *P. aeruginosa* ATCC 27853, *C. albicans*, and *C. tropicalis* demonstrated that while some compounds have 200 and 800 μg/mL MIC values against Gram-positive bacteria (*E. faecalis* and *S. aureus*), all compounds have no antimicrobial activity against Gram-negative bacteria (*E. coli* and *P. aeruginosa*).^53–55^ In a previous study, it was demonstrated that benzimidazole–cobalt complexes have better antimicrobial activity against *P. aeruginosa* and *S. aureus* than *E. coli* and the study showed that the MIC values for *E. coli* are higher than the MIC values for *Candida* species.^53^ It has also been reported that Gram-negative bacteria are more resistant than Gram-positive bacteria to the cobalt­(II) complexes containing 1-benzylbenzimidazoles.^56^ Furthermore, the cationic bis-benzimidazole-silver­(I) complexes were shown to have higher antifungal activity than their antibacterial effect.^57^

**Table 3:** MIC Values (μg/mL) of Benzimidazole-Co­(II) and-Zn­(II) Complexes against Microorganisms (SD of the Mean MIC Values Was 0)

	*E. coli*	*P. aeruginosa*	*S. aureus*	*C. albicans*	*C. tropicalis*
**Compound 1**	>100	50	>100	>100	>100
**Compound 2**	>100	12.5	50	25	25
**Compound 3**	>100	>100	>100	>100	>100
**Compound 4**	>100	100	100	25	25
**Compound 5**	>100	100	>100	12.5	12.5
**Compound 6**	>100	50	100	12.5	12.5
Gentamicin	0.78	0.39	3.125		
Fluconazole				0.39	0.39

## 4 Conclusion

In this study, the cytotoxic and antimicrobial properties of newly synthesized derivatives of benzimidazole-metal complexes bearing methyl and nitro substituents at position 5(6) were investigated. The results demonstrated that cobalt complexes containing methyl groups at the para position of the benzyl group have higher cytotoxic activity against the A549 cancer cell line than the BEAS-2B healthy cell line. While Co­(II) has a partially filled 3d^7^ high-spin electronic configuration, Zn­(II) has a closed-shell 3d^10^ configuration. Because of the paramagnetic and redox-active nature of Co­(II), its complexes may participate in ligand-exchange reactions, transient redox processes, and interactions with intracellular biomolecules more readily than Zn­(II) complexes. These provide higher intracellular reactivity (ROS generation, mitochondrial membrane destabilization, and interactions with DNA/proteins). This is consistent with the increased caspase-3 activity and elevated levels of p53 expression observed in this study. Compounds **4** and **5** have high cytotoxic activity on the A549 cancer cell line, whereas the markedly lower cytotoxicity against the BEAS-2B healthy cell line highlights their potential as promising candidates for anticancer drug development. Metal complexes also showed higher cytotoxic activity than ligand **1**. Furthermore, some of these compounds showed high antimicrobial activity, suggesting their potential as an antimicrobial agent against pathogens. As a result, these benzimidazole-metal complexes may be a scaffold for new anticancer and antimicrobial drug candidates.

## Supplementary Material



## Data Availability

Information will be provided upon request.

## Notes

^1^H NMR, ^13^C NMR, FTIR, and UV–vis spectra for all synthesized compounds (PDF)
