# Supplementary material for: Anticancer and Antimicrobial Activities of New Cobalt and Zinc Complex-Derived Benzimidazole Containing Nitro or Methyl Groups
Source: ACS Omega. 2026 Feb 9;11(7):11319–27. doi: 10.1021/acsomega.5c08353 (PMC12946976; doi:10.1021/acsomega.5c08353)
Supplement: Supplementary file 1 [file ao5c08353_si_001.pdf]

Anticancer and antimicrobial activities of new cobalt and zinc complexes derived benzimidazole containing nitro or methyl groups

Ozgur Yilmaz[1,*], Elif Apohan[1*], Ozfer Yesilada[1], Ülkü Yılmaz[2], Hasan Küçükbay[3]

[1]Inonu University, Art and Science Faculty, Department of Biology, Malatya, TURKEY

[2] Malatya Turgut Özal University, Engineering and Natural Sciences Faculty, Department of Engineering Basic Sciences Malatya, TURKEY

[3] Inonu University, Art and Science Faculty, Department of Chemistry, Malatya, TURKEY

^{1}H, ^{13}C NMR, FT-IR, and UV-Vis Spectra of New Compounds

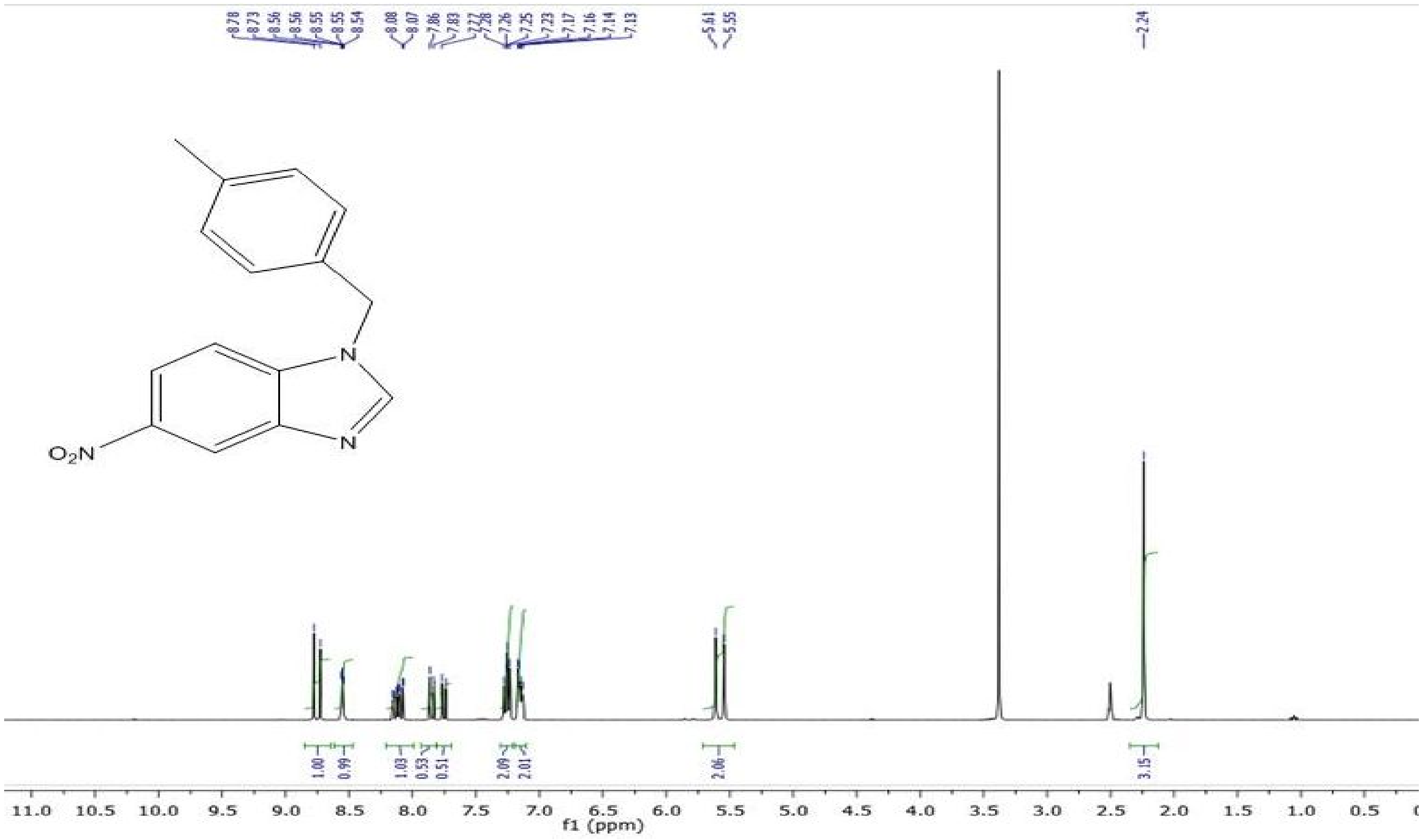

Figure S1. ^{1}H NMR spectrum of compound **1**

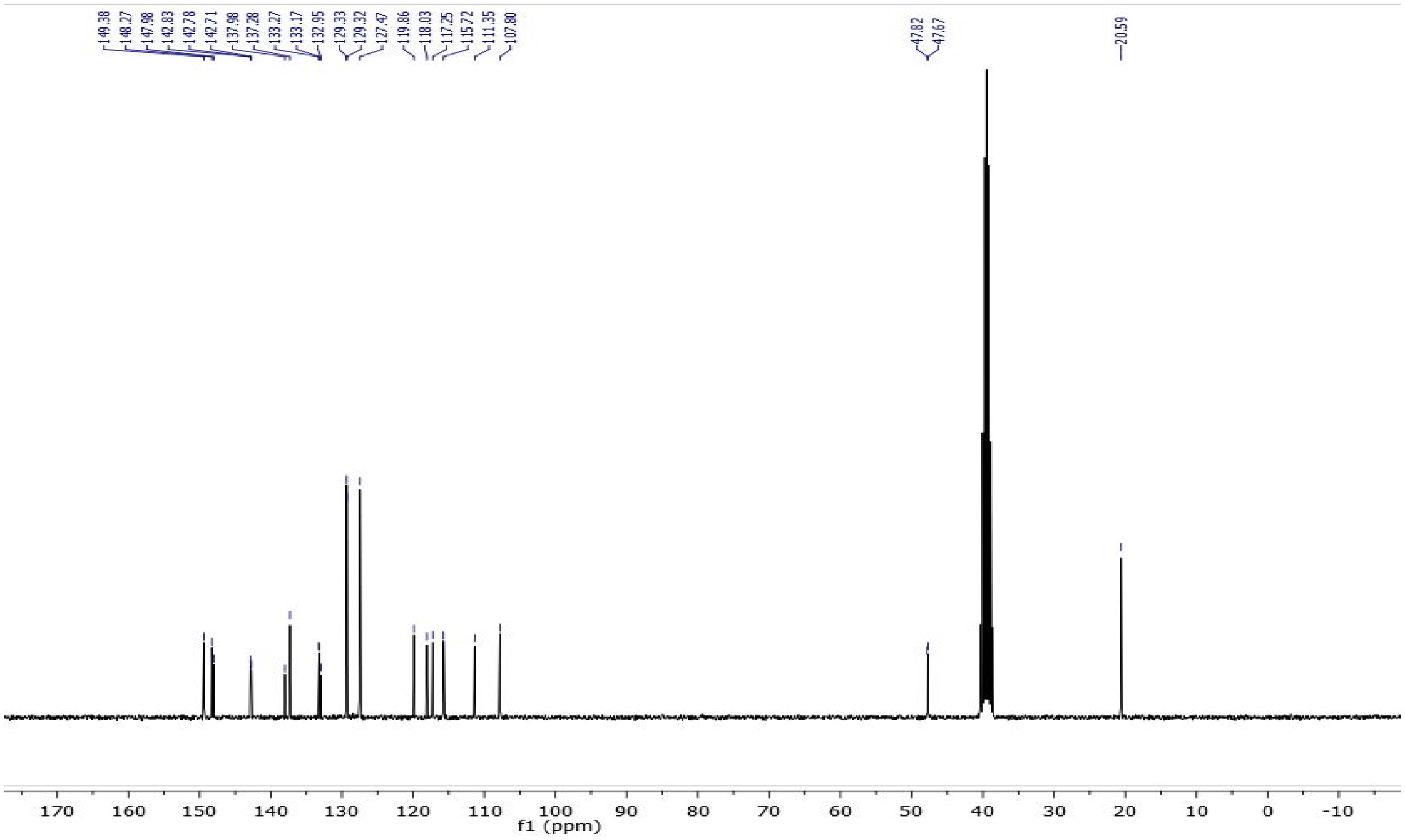

Figure S2. ^{13}C NMR spectrum of compound **1**

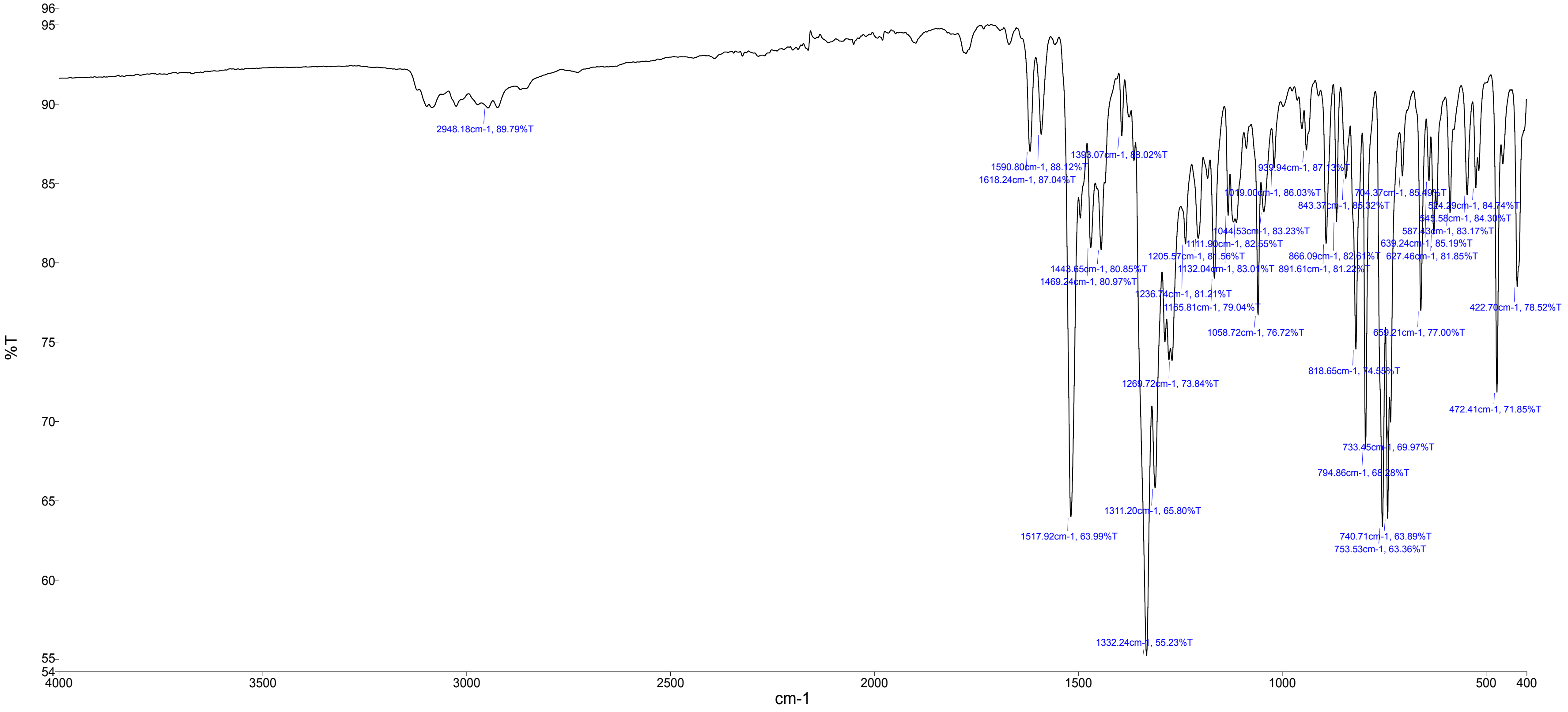

Figure S3. FT-IR spectrum of compound **1**

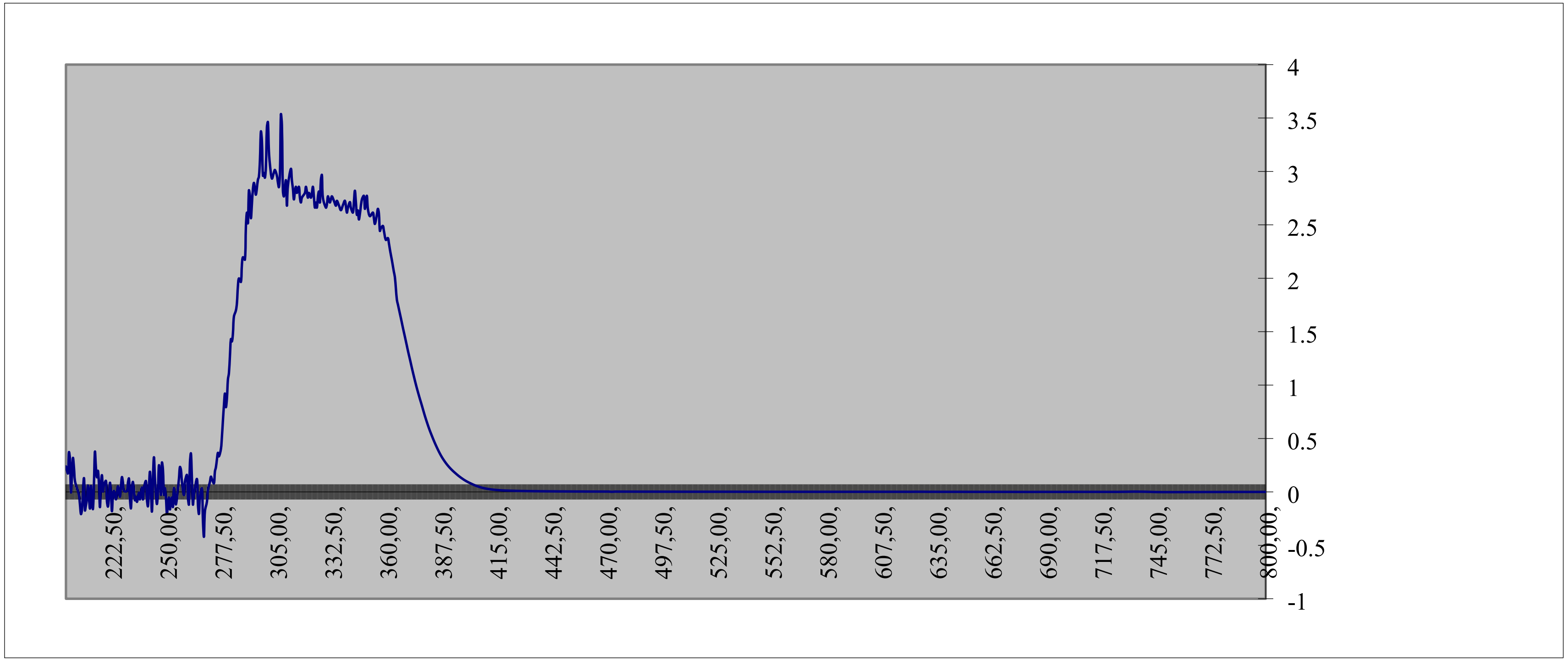

Figure S4. UV-Vis spectrum of compound **1**

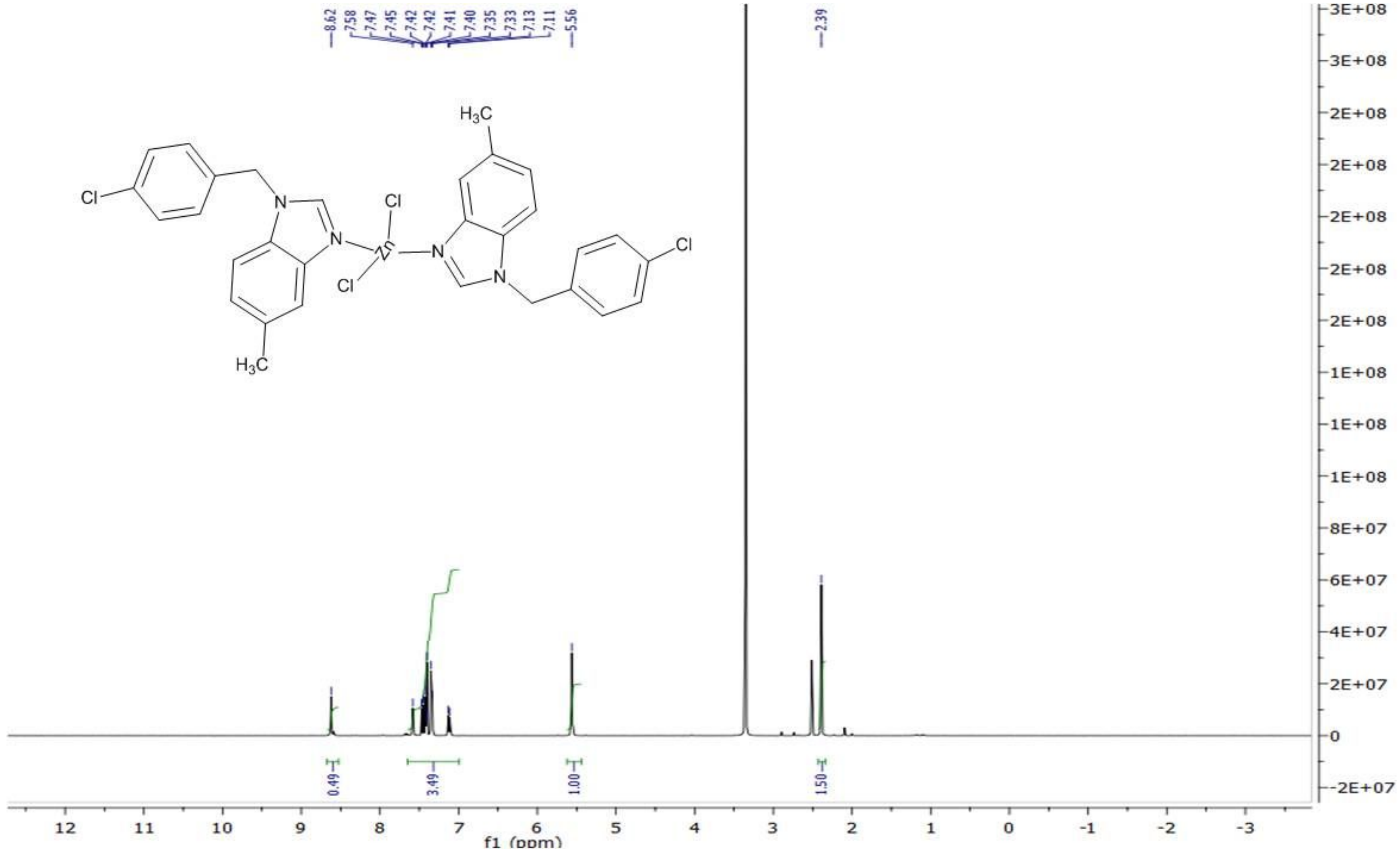

Figure S5. ^{1}H NMR spectrum of compound **2**

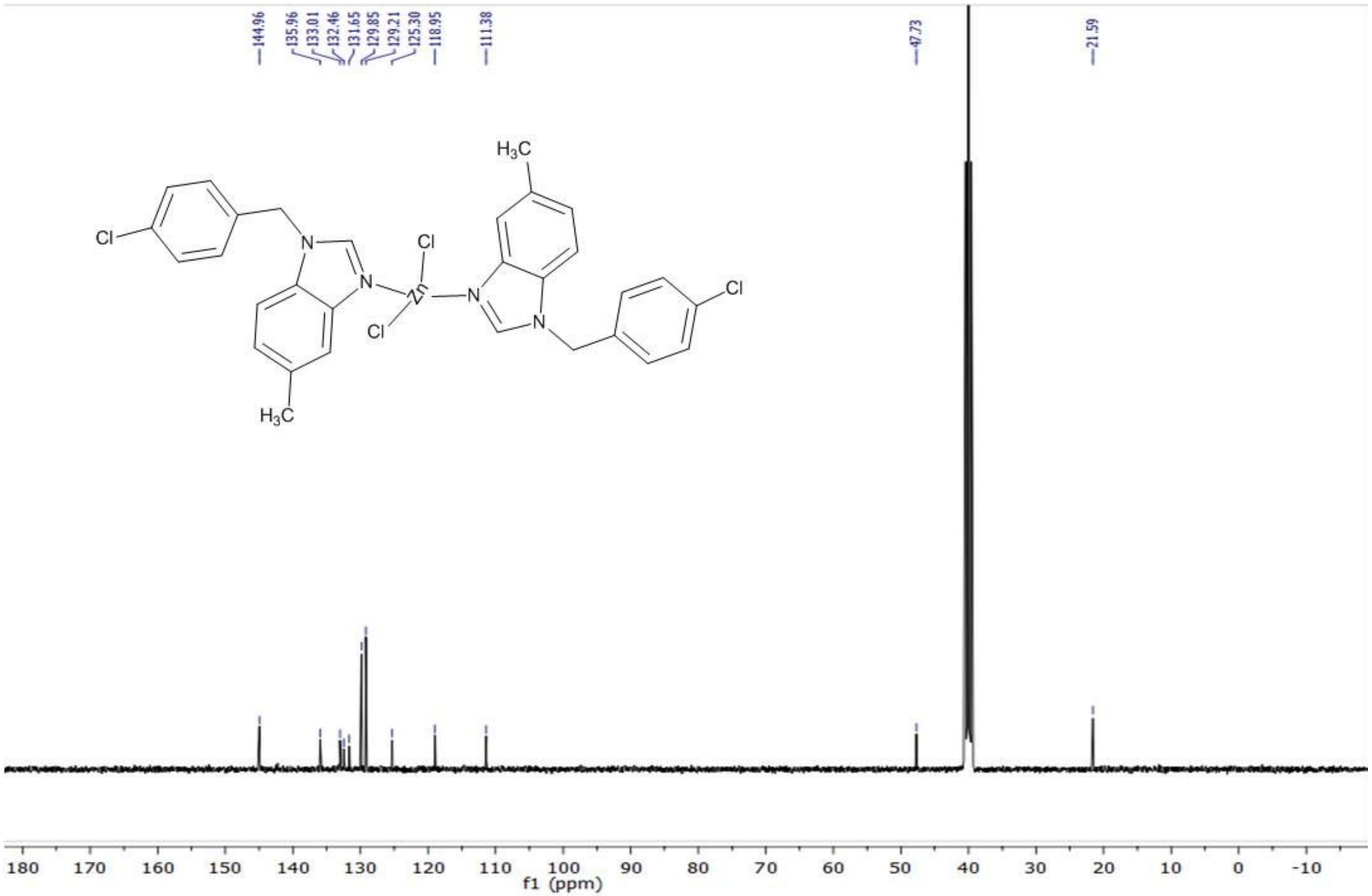

Figure S6. ^{13}C NMR spectrum of compound **2**

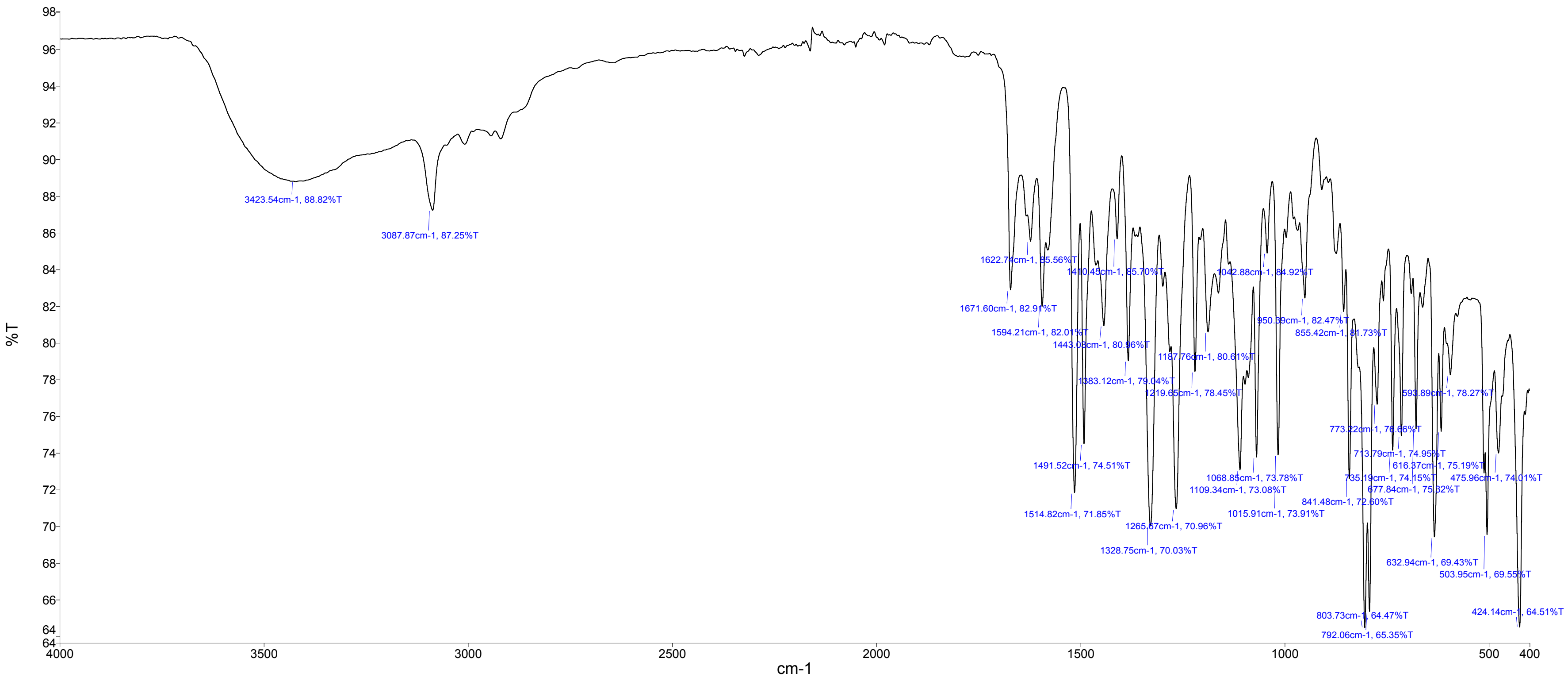

Figure S7. FT-IR spectrum of compound **2**

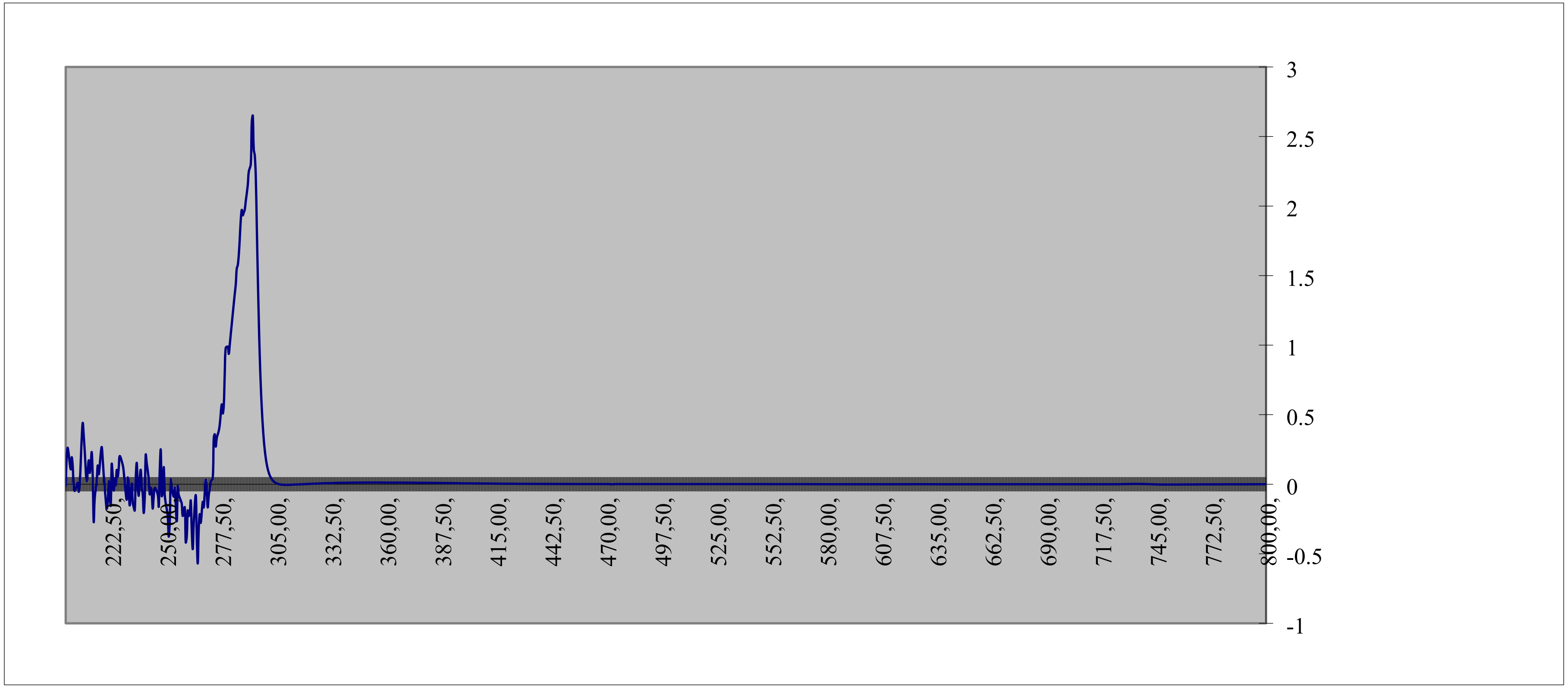

Figure S8. UV-Vis spectrum of compound **2**

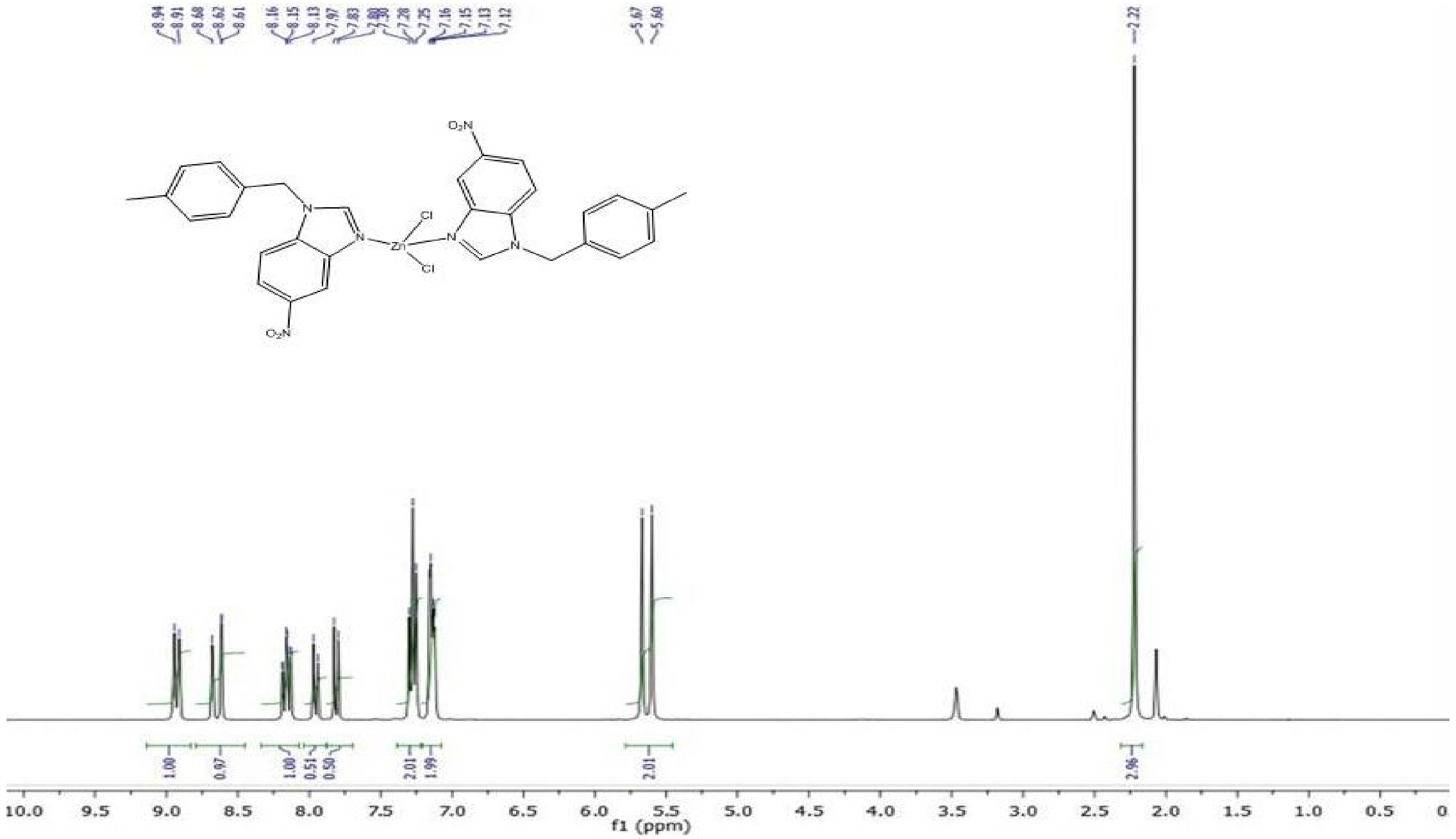

Figure S9. ^{1}H NMR spectrum of compound **3**

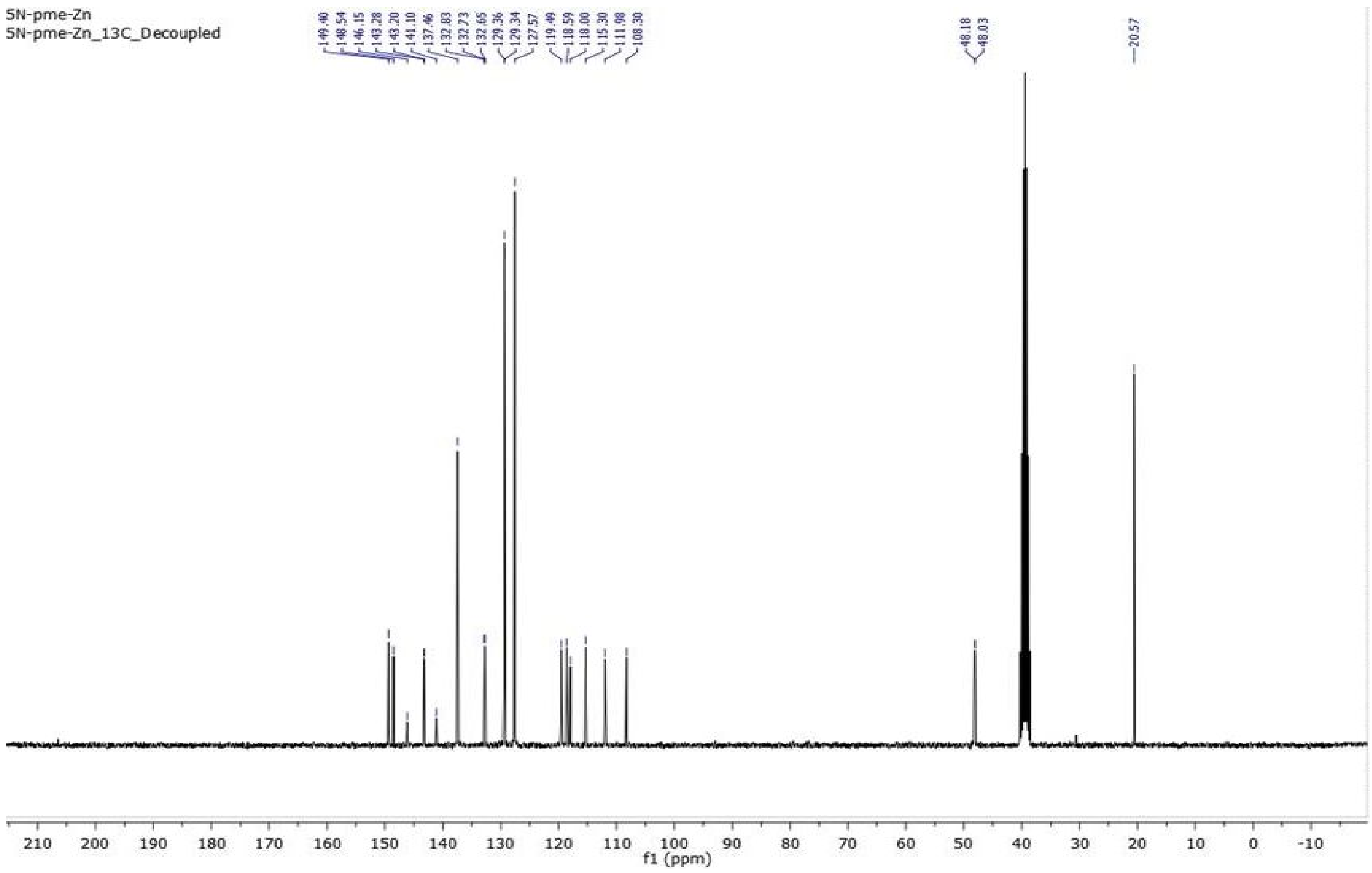

Figure S10. ^{13}C NMR spectrum of compound **3**

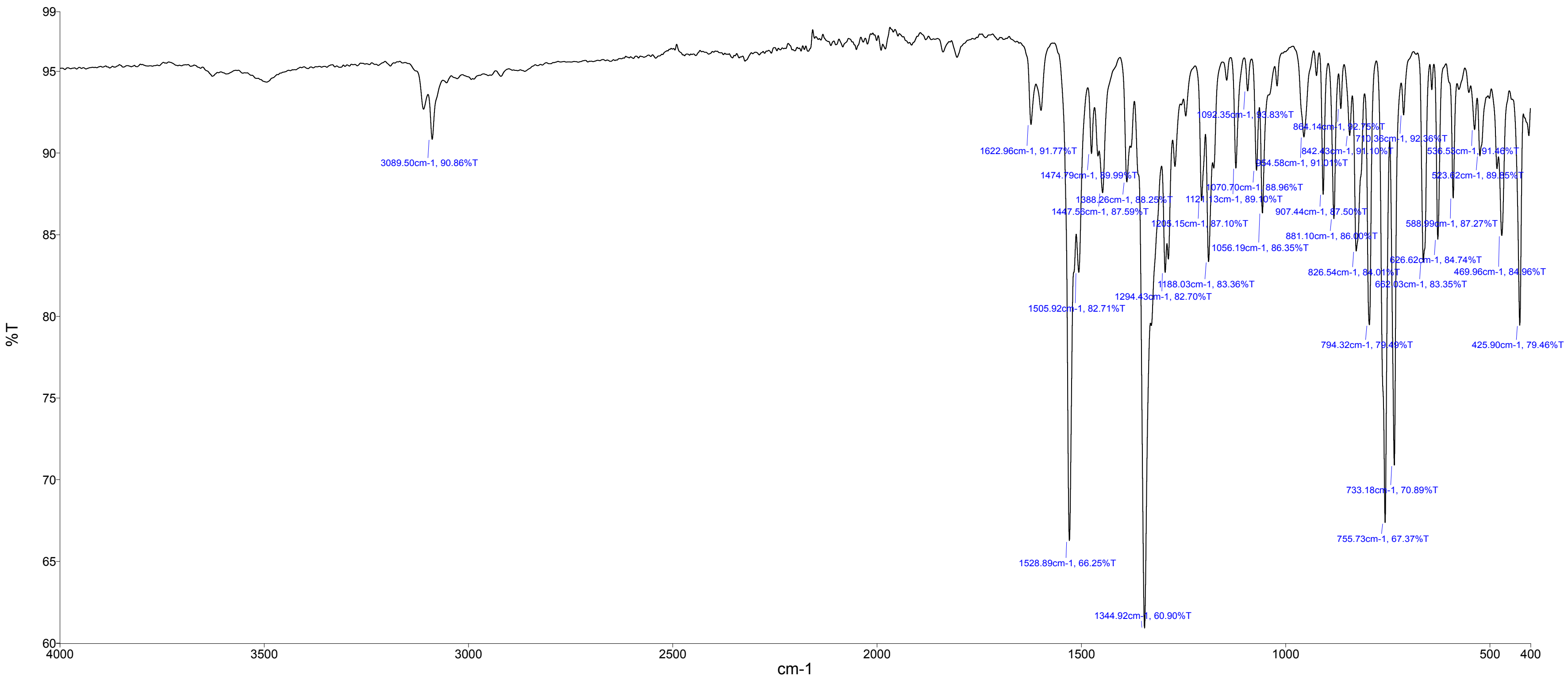

Figure S11. FT-IR spectrum of compound **3**

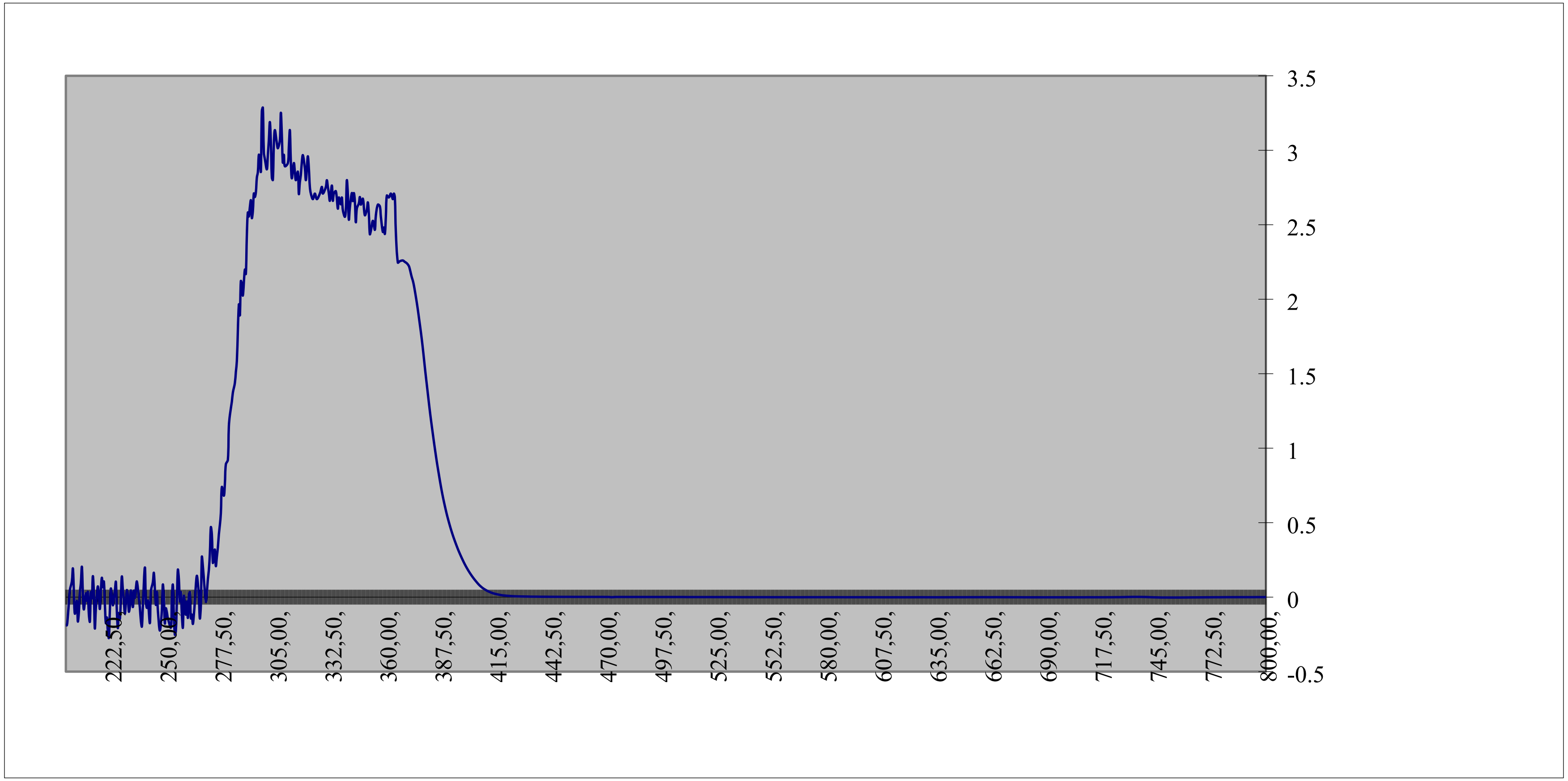

Figure S12. UV-Vis spectrum of compound **3**

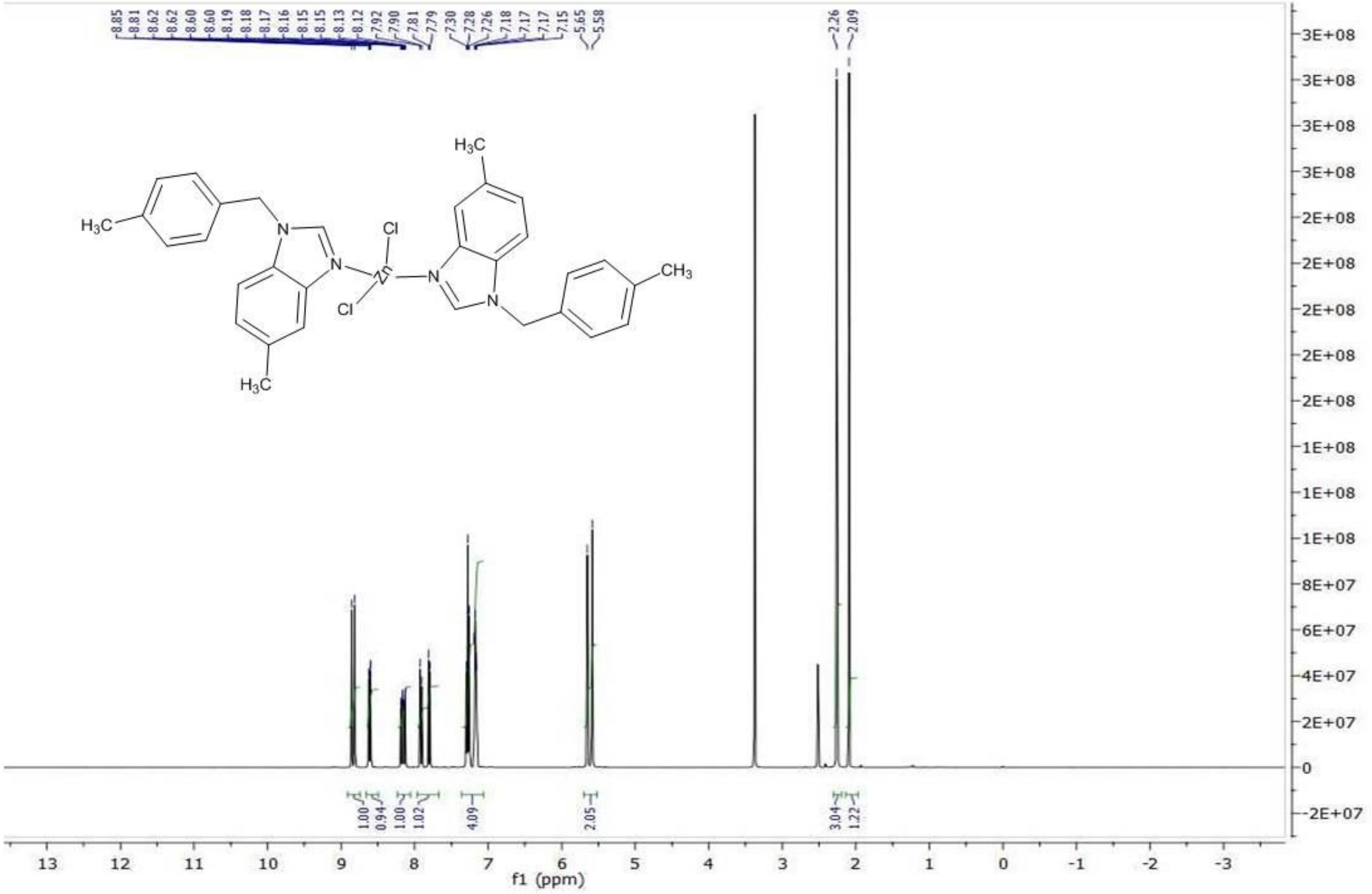

Figure S13. ^{1}H NMR spectrum of compound **4**

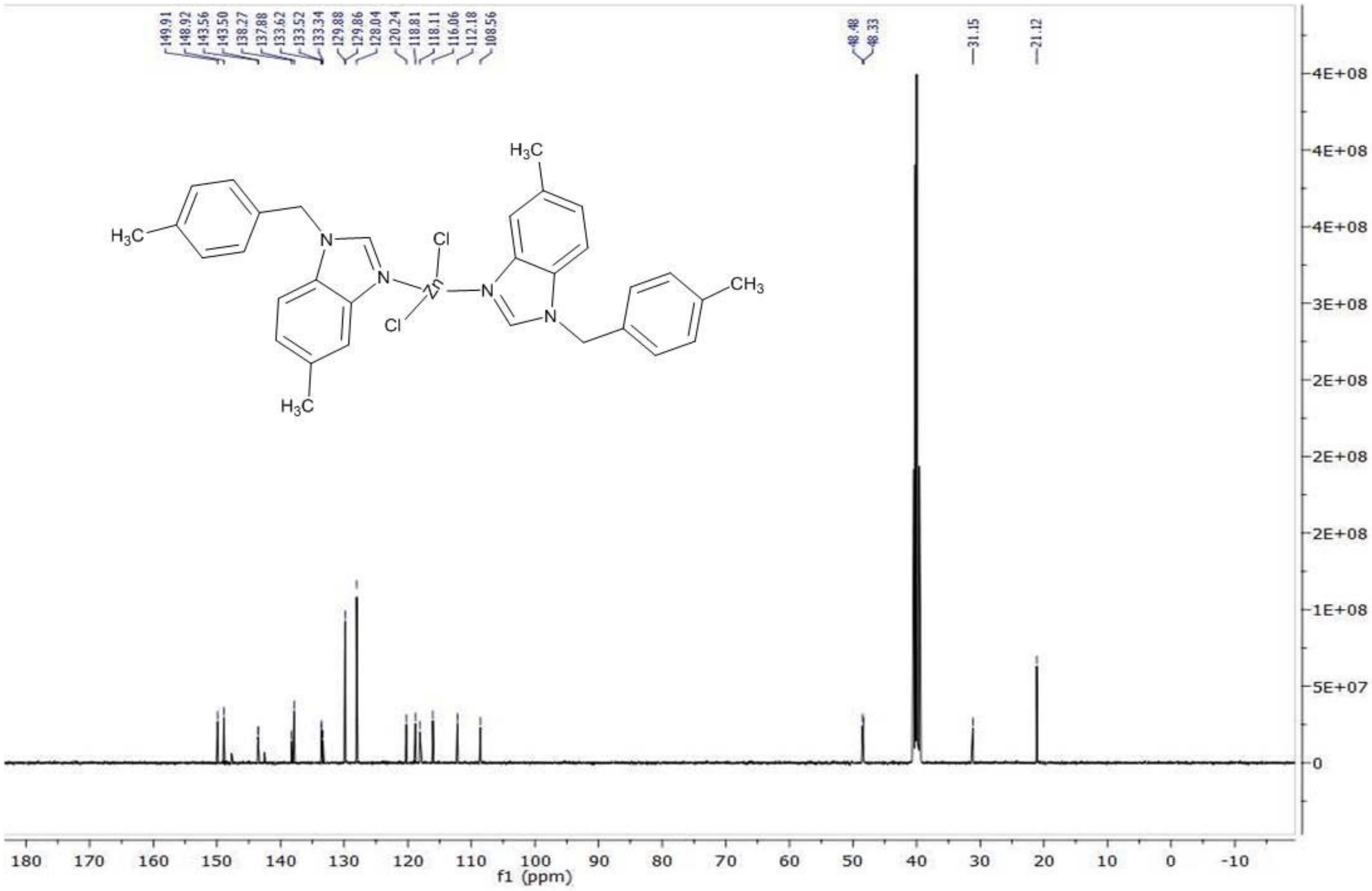

Figure S14. ^{13}C NMR spectrum of compound **4**

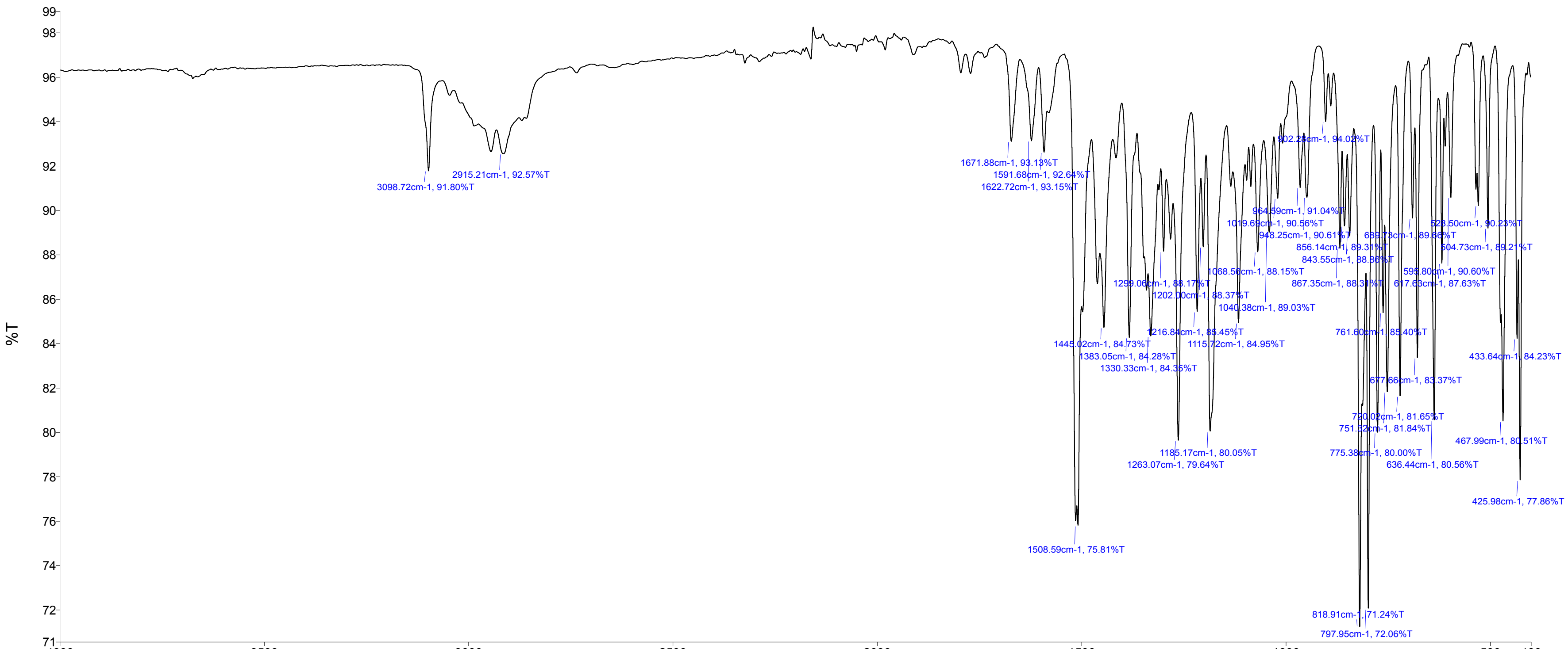

Figure S15. FT-IR spectrum of compound **4**

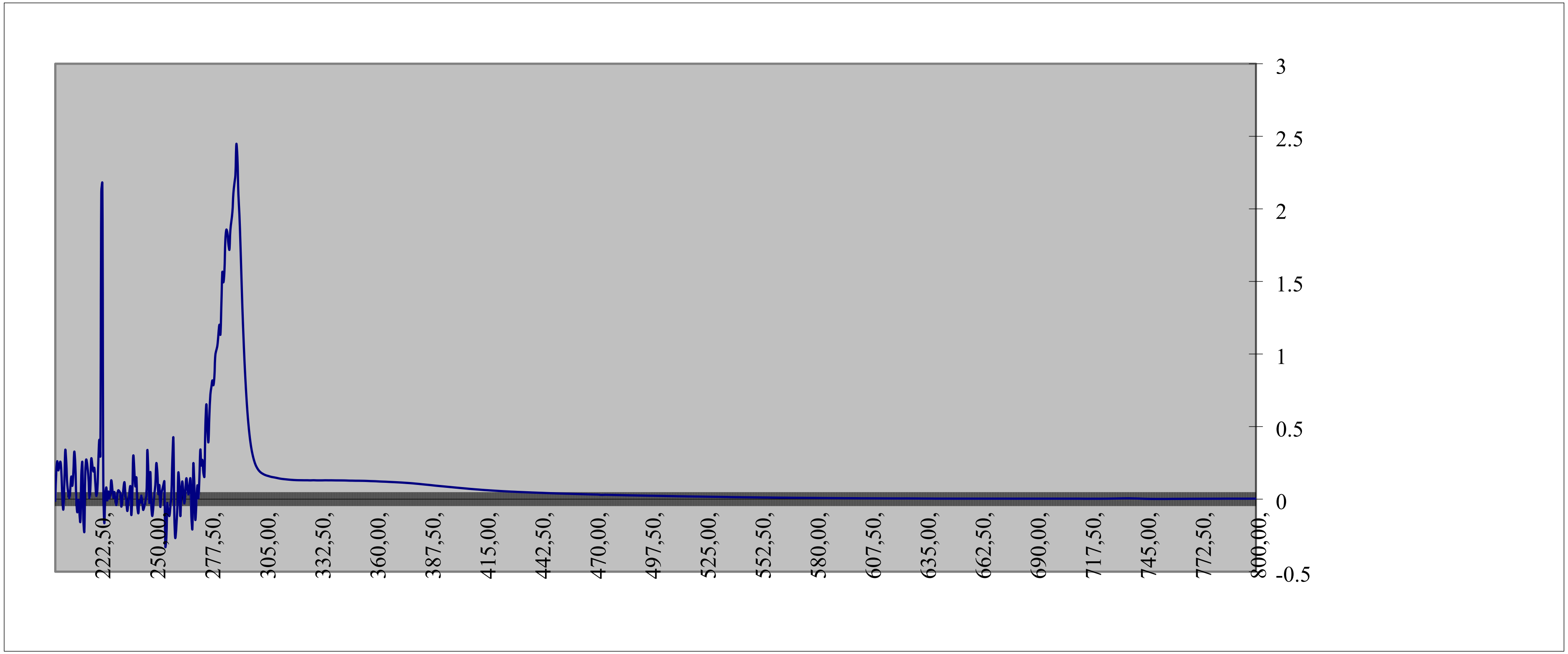

Figure S16. UV-Vis spectrum of compound **4**

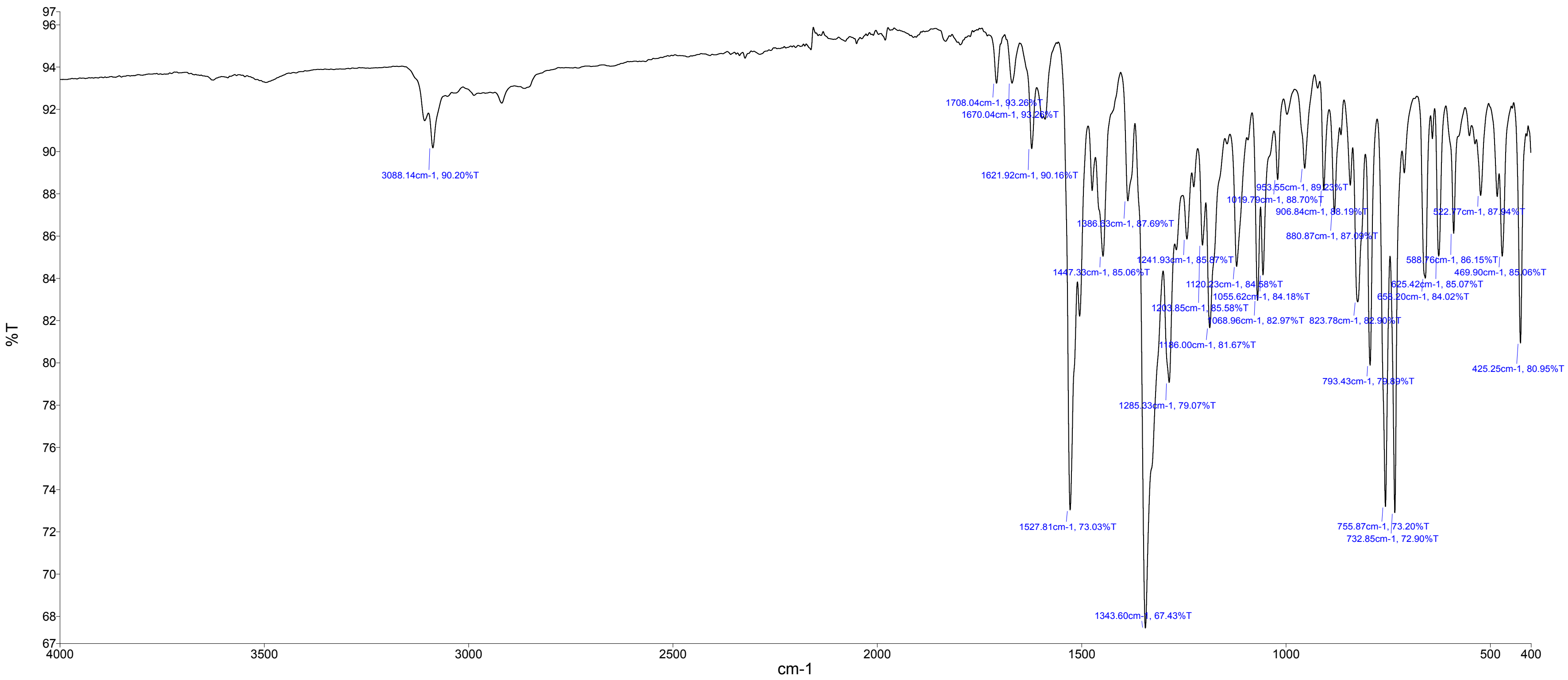

Figure S17. FT-IR spectrum of compound **5**

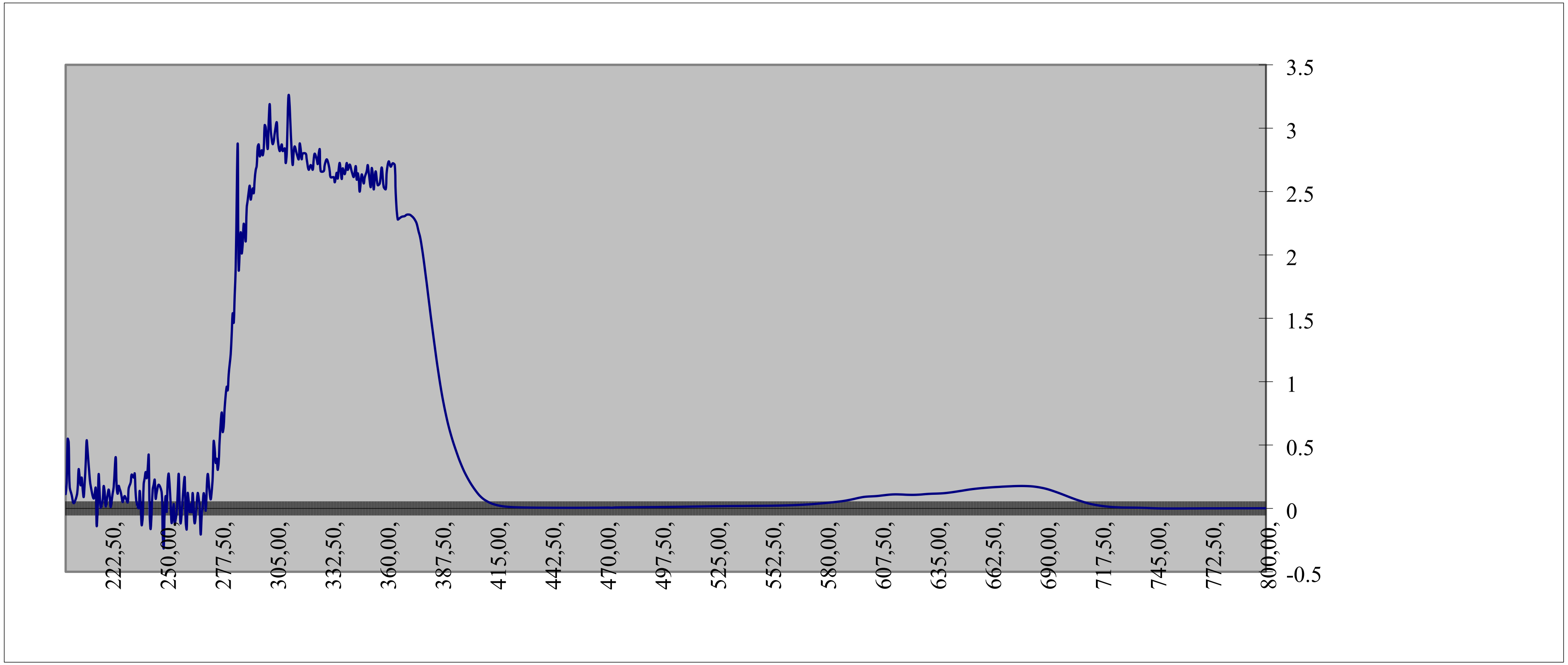

Figure S18. UV-Vis spectrum of compound **5**

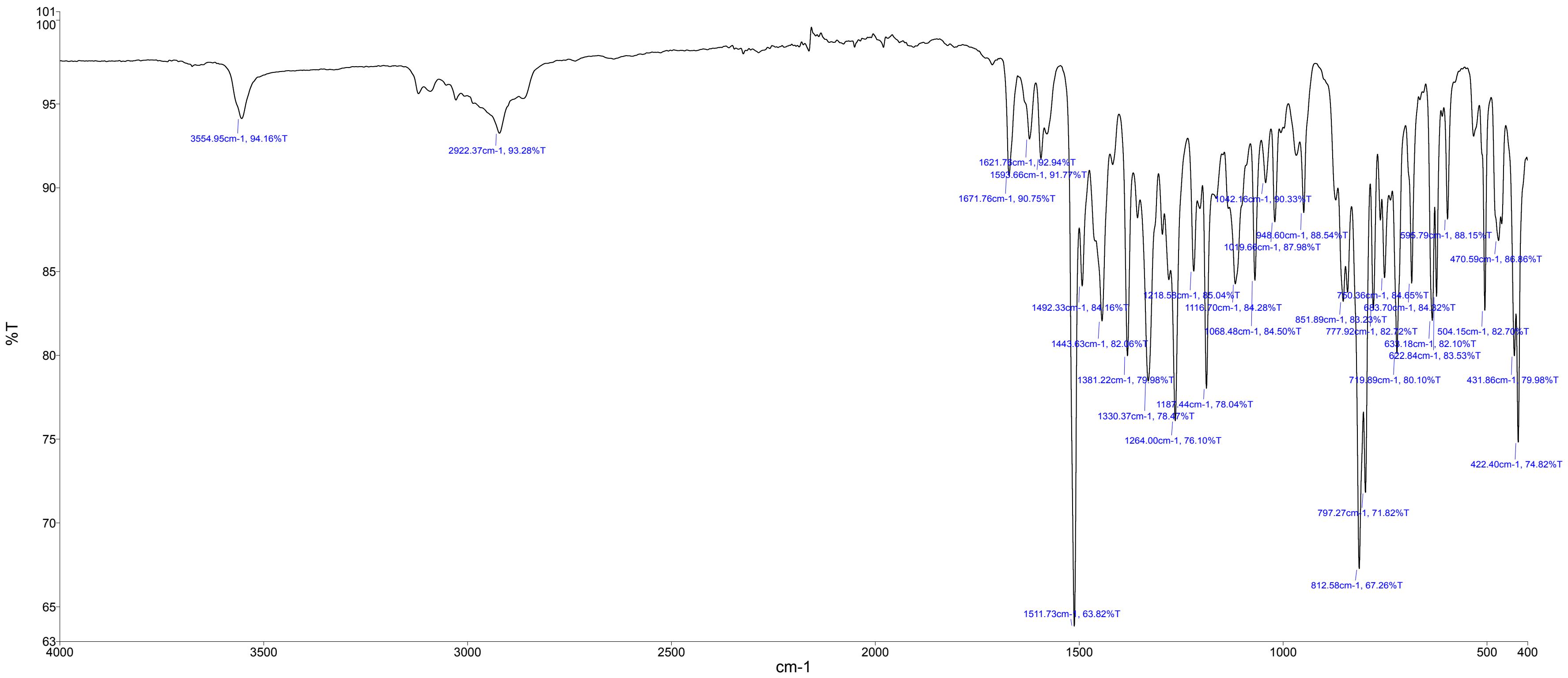

Figure S19. FT-IR spectrum of compound **6**

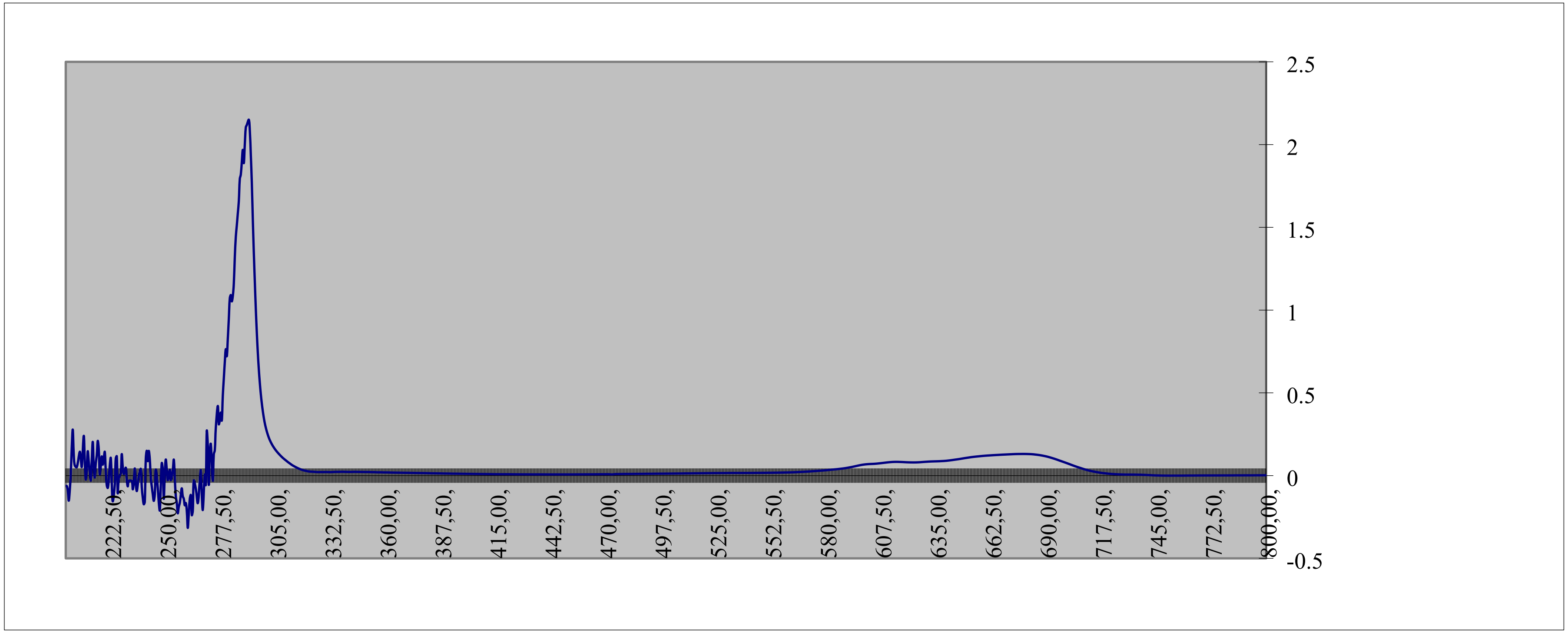

Figure S20. UV-Vis spectrum of compound **6**
